# Toxic Alerts of Endocrine Disruption Revealed by Explainable Artificial Intelligence

**DOI:** 10.1021/envhealth.4c00218

**Published:** 2025-01-27

**Authors:** Lucca Caiaffa Santos Rosa, Mariam Sarhan, Andre Silva Pimentel

**Affiliations:** Departamento de Química, Pontifícia Universidade Católica do Rio de Janeiro, Rio de Janeiro, RJ 22453-900, Brazil

**Keywords:** regulatory decision-making, environmental and human health risks, chemical exposure, interpretability, explainability, emerging contaminants

## Abstract

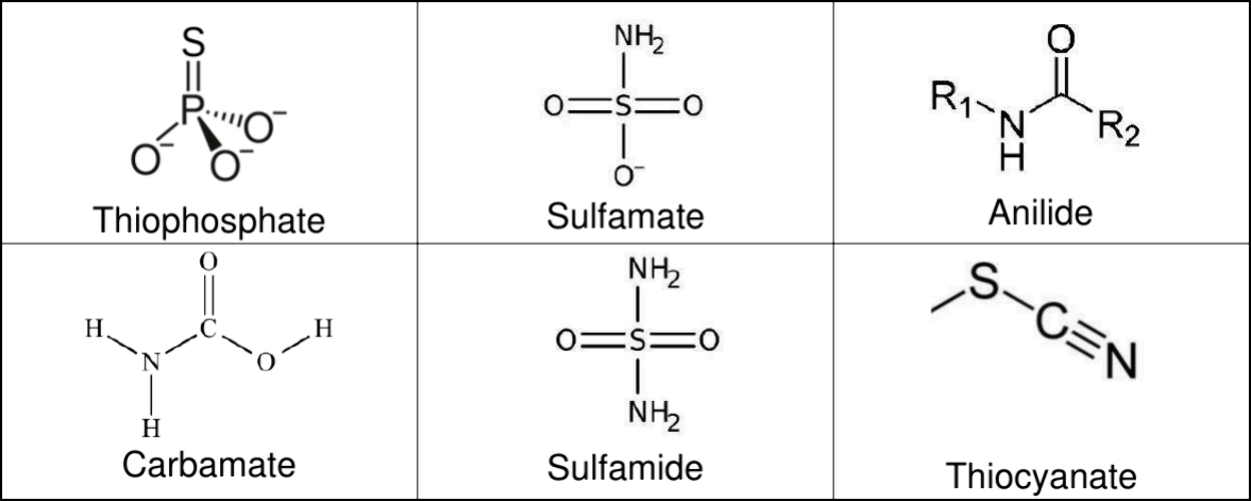

The local interpretable model-agnostic explanation method was used to unveil substructures (toxic alerts) that cause endocrine disruption in chemical compounds using machine learning models. The random forest classifier was applied to build explainable models with the TOX21 data sets after data curation. Using these models applied to the EDC and EDKB-FDA data sets, the substructures that cause endocrine disruption in chemical compounds were unveiled, providing stable, more specific, and consistent explanations, which are essential for trust and acceptance of the findings, mainly due to the difficulty of finding relevant experimental evidence for different receptors (androgen, estrogen, aryl hydrocarbon, aromatase, and peroxisome proliferator-activated receptors). This approach is significant because of its contribution to the interpretability of explainable machine learning algorithms, particularly in the context of unveiling substructures associated with endocrine disruption in five targets (androgen receptor, estrogen receptor, aryl hydrocarbon receptors, aromatase receptors, and peroxisome proliferator-activated receptors), thereby advancing the relevant field of environmental toxicology, where a careful evaluation of the potential risks of exposure to new compounds is needed. The specific substructures thiophosphate, sulfamate, anilide, carbamate, sulfamide, and thiocyanate are presented as toxic alerts that cause endocrine disruption to better understand their potential risks and adverse effects on human health and the environment.

## Introduction

Endocrine disruption is a phenomenon that has significant implications for human health, relying on the subtle interplay between chemical compounds and the endocrine system.^1^ Understanding endocrine disruption is a crucial challenge in the field of chemical toxicology, where the effects of molecular interactions resonate in the environment and affect health.^1,2^ The exploration of the substructures that render chemical compounds potent endocrine disruptors is essential for comprehending the molecular interactions between the endocrine disruptors and receptors.^3,4^

Machine learning explainer models,^5^ finely tuned to capture the complexities of chemical structures, introduce a new perspective to the challenge of finding the correlation between substructures and endocrine disruption. By analyzing massive data sets that include diverse chemical compounds and their corresponding endocrine disruption outcomes, these models discern patterns and substructures that elude conventional approaches. The identification of molecular motifs that act as potent kernels of endocrine disruption is the key to unraveling the significance of these substructures, shedding light on the molecular fingerprints that underpin the toxicological impact of diverse chemical compounds. Therefore, the method adopted here promotes a deeper understanding of the unknown substructures that contribute to endocrine disruption.

Explainable machine learning^5^ has emerged as an invaluable tool in toxicology, offering transformative capabilities in understanding the relationships between chemical structures and biological responses.^6−13^ The interpretability of explainable machine learning models allows us to unlock it and gain profound insights into the substructures that drive endocrine disruption.^3,4,14^ Each step from data curation and cross-validation to random tree model training, statistical analysis,^8,9,12,13^ and local interpretable model-agnostic explanations (LIME),^15^ is undertaken meticulously to ensure robustness and reliability in identifying patterns, decoding relationships, and elucidating the specific substructures that correlate with endocrine disruption.^14^

The groundwork laid in this study aims to uncover key insights and answer an important question: Which substructures distinguish endocrine disruptors at the molecular level?^16^ Our findings promise to reshape our understanding of these chemical compounds, providing a nuanced and deeper perspective on the substructures that make them potent endocrine disruptors.^3,4^ The purpose of this study is to identify these substructures and classify them as interacting with androgen receptors (AR), estrogen receptors (ER), aryl hydrocarbon receptors (AhR), aromatase receptors (ARO), and peroxisome proliferator-activated receptors (PPAR) using random forest (RF) classifier models. The machine learning model is trained and validated using the meticulously curated TOX21^17^ data set and tested with the EDC^18^ and EDKB-FDA^19^ data sets. The quality of these models is assessed using metrics such as precision, recall, F1, Matthew Correlation Coefficient (MCC), and accuracy scores. The machine learning models are interpreted using the local interpretable model-agnostic explainer LIME,^15^ which identifies the most relevant substructures that cause endocrine disruption. The novelty of our work lies in providing an explained, comprehensive list of more specific toxic alerts relevant for endocrine disruption that surpasses the existing knowledge in five tasks as compared with literature^4,20−23^ from experimental and explainable methods so far.

## Methodology

The Python packages DeepChem (version 2.7.1.), RDKit (version rdkit-2023.9.1), and LIME (version lime-0.2.0.1) were installed on a Google Colaboratory platform. Other Python auxiliary packages such as mols2grid (version mols2grid-2.0.0), Matplotlib (version 3.7.1), Scikit-learn (version 1.2.2), pandas (version 1.5.3), Numpy (version 1.23.5), and IPython (version 8.19.0) were also installed.

The TOX21,^17^ EDC,^18^ and EDKB-FDA^19^ data sets were carefully curated for training, validating, and testing machine learning models for endocrine disruption. The TOX21 data set includes the SMILES representation and binary labels (0 for inactive and 1 for active molecules) for the activity of 8014 compounds on AR, ER, AhR, ARO, and PPAR. We used DeepChem to implement various data splitters, transformers, featurizers, machine learning classifier models, and infrastructure to create a model for classifying compounds with endocrine disruption ability. The TOX21^17^ data set was featurized using 1024 extended-connectivity fingerprints (ECFPs), a type of topological fingerprint for molecular characterization. As TOX21 data set is imbalanced, we treated it with the resample method using the Scikit-learn library to create two different dataframes of majority and minority classes, and upsampled the minority class. The featurized data set was then randomly split (80/20 split) for training and validation using the 5-fold cross-validation method for the RF classifier models. It is important to note that the model is randomly generated for each run, so the results may represent the expected diversity. We splitted 20% of the TOX21 data set for internal 5-fold cross validation. Then, the external validation was performed using the EDC and EDKB-FDA data sets.

Initially, RF classifier models were used from the Scikit-learn library. The hyperparameters of these models were optimized, specifically, the number of estimators (50 and 500) and maximum depth (1 and 20) of the trees, using the randomized search CV method and the ROC-AUC metric. The best models were subsequently used in the next steps with the training and validation data sets, evaluating the area under the ROC curve (ROC-AUC metric). The results were assessed using the confusion matrix, accuracy, precision, F1, MCC, and recall scores.

LIME^15^ was utilized to explain the predictions made by the model using the training and validation data sets (TOX21), and test data sets (EDC and EDKB-FDA) using the statistical analysis. In the classification mode, the model explainer was employed with categorical and feature names using 1024 circular fingerprints to represent the training data set and feature names. Then, the model predicted and explained the validation data set using 100 features, resulting in a dictionary that maps the fingerprint index to the list of SMILES strings that activated that fingerprint, which contains their respective substructure weights that are important for endocrine disruption, allowing us to understand why a molecule is classified as such. Each molecule in the validation data set was evaluated to explain why the model predicted it as an endocrine disruptor or not. Disrupting molecules that were correctly predicted as endocrine disruptors in the validation data set were filtered out by applying a threshold of 0.8 to use only the most important disruptors. Therefore, the explainer identified the most sensitive features to the prediction by analyzing the elements in the fingerprint that correspond to one or more fragments. Then, these findings were stored to be mapped and visualized. LIME assigned weights to indicate the contribution of a fragment in a molecule to the prediction, ranging from 0 (no disruption) to −1 (negative impact on disruption) or +1 (positive impact on disruption). Therefore, the sum of these weights for each molecule does not necessarily equal 1, but still indicates the contribution of a fragment to the prediction. Thus, the model classified molecules as “endocrine disruptor” or “nonendocrine disruptor” by separately summing all the positive and negative contributions of its fragments. If the sum of the positive weights is greater than the sum of the negative weights, the molecule is classified as an “endocrine disruptor”. Lastly, the classification data of each active molecule for endocrine disruption was displayed with the number of occurrences and total weight of contribution of each substructure in the endocrine disruption prediction. Fragments with small or negative weights were filtered out to enhance the interpretability of the results, focusing on the substructure that strongly enables endocrine disruption. The RDKit resources were applied to highlight the substructures, and the visualization of them in the associated compounds was rendered by the mols2grid and Pandas libraries.

## Results and Discussion

### Data Curation

The TOX21 data set originally had 8014 chemical compounds distributed in five tasks of nuclear receptors: AR, ER, AhR, ARO, and PPAR. Then, for each receptor, to eliminate repeated instances for each compound, the Tanimoto similarity coefficients were calculated using Morgan fingerprints. We dropped compounds with coefficients equal to 1 from the data set, only keeping the first occurrence of the compound. Additionally, the repeated chemical compounds with different labels (0 for one compound and 1 for the same compound) were both dropped to avoid inconsistence. All multiple occurrences were also dropped, keeping only the first occurrence for each repeated molecule with matching labels. Consequently, the five subdata sets with each of the five tasks were reduced to 6670, 5627, 5964, 5268, and 5909 compounds for the receptors AR, ER, AhR, ARO, and PPAR, respectively. The EDC and EDKB-FDA test data sets had originally 1107 and 632 chemical compounds, respectively. They were also curated using the same procedure presented above. Thus, the remaining number of compounds in the EDC and EDKB-FDA testing data sets was 1077 and 287 after curation, respectively. Table 1 shows the numbers of initial and final compounds with activity data per assay and number of duplicates and multiple occurrences for each task.

**Table 1:** Numbers of Initial and Final Compounds with Activity Data Per Assay, Compounds with No Data, and Number of Duplicates and Multiple Occurrences for Each Task

Task	# of initial compounds	Compounds with no data	Compounds with unmatched data	Duplicates/Multiple occurrences	Salts/Complexes	# of final compounds
AR	8014	575	71	506/495	219	6670
ER	8014	1698	431	326/315	0	5627
AhR	8014	1323	171	446/455	217	5964
ARO	8014	2074	312	373/387	216	5268
PPAR	8014	1431	218	418/436	217	5909
EDC	1107	0	0	36/15	0	1077
EDKB	632	0	0	350/199	0	286

### Building the Best Classifier Model

Previously, we tested Deep Residual Network (DRN) implemented in the DeepChem package in previous studies in our group (not shown here).^14,24,25^ From our experience, also demonstrated here, RF and ET give equivalent results using the metrics mentioned earlier, however GNB and DRN are inferior compared to RF and ET. In addition, DRN is much slower method than RF, ET, and GNB, so we decided to use RF model because it is a traditional and robust ML model. The ROC-AUC, precision, recall, F1, accuracy, MCC and Cohen′s Kappa scores for Extra Trees (ET), Random Forest (RF), and Gaussian Naïve Bayes (GNB) classifier models for the train and validation data sets of endocrine disruption found in TOX21 data set are presented in Supporting Information (Table S1).

### Treating the Imbalanced TOX21 Data Set

We performed oversampling before 80/20 splitting to tune hyperparameters and optimize the model using internal 5-fold cross validation (See below). We also performed several combinations of oversampling and downsampling methods before and after splitting to find the best approach. We found that the best approach is oversampling the minority class before the splitting. First, we used internal validation to evaluate the performance on a data set that is often derived from the same population or distribution as the training data. This approach usually yields overfitting, particularly when imbalanced classes are resampled, leading to optimistic performance metrics. Then, we performed two external validations using the EDC and EDKB-FDA data sets as presented in Supporting Information (Table S2). These external validations tested the model on two entirely independent data sets that were not involved in model development. This step was crucial for assessing the generalizability of the model. This approach gives insights into how well the model fits the data used during training. And, the 5-fold cross validation ensures that the model is tested against various portions of the data, improving reliability. This ensures that the model performs well on unseen data, reflecting its ability to make accurate predictions in real-world scenarios and detects whether the model is overly influenced by artifacts or biases specific to the internal data set. Finally, this approach prevents data leakage and overlap of training and testing data, which can lead to artificially inflated performance metrics.

### Hyperparameter Optimization and Statistical Analysis

*k*-fold cross-validation involves dividing the data set into *k* subsets (folds) of equal size, using 1-fold for training and the remaining one for validation, iteratively cycling through all folds. This ensures that every data point is used for both training and validation. A smaller k (e.g., *k* = 3) increases variance in the validation results because fewer data points are used for training, and the folds may not fully represent the data set’s diversity. A larger *k* (e.g., *k* = 10) provides more stable and reliable estimates of model performance but increases computational cost. Model robustness refers to the model’s consistency and stability across different folds and unseen data. If ROC-AUC fluctuates significantly with increasing *k*, the model may rely on specific data patterns rather than learning generalizable features. By analyzing ROC-AUC metrics under different *k*, it is possible to check for consistency across folds and evaluate generalization. If ROC-AUC remains stable across all folds and across different *k* values, the model is likely robust to variations in training data subsets. However, significant variability in ROC-AUC across folds may indicate sensitivity to data splits, suggesting the model is not robust. If ROC-AUC scores drop significantly when transitioning from training to validation folds, it suggests the model may be overfitting (performing well on training data but poorly on validation data). Overfitting occurs when the model captures noise or specifics of the training data that do not generalize to unseen data. To detect overfitting using *k*-fold validation and ROC-AUC, it is important to compare the ROC-AUC scores of the training folds to those of the validation folds. Large discrepancies (training AUC much higher than validation AUC) indicate overfitting. Smaller k values (e.g., *k* = 3) can exaggerate overfitting, as the training set is smaller, and the model is less likely to generalize well to the validation fold. However, larger *k* values (e.g., *k* = 10) mitigate this risk by providing a larger training set and more representative validation results, offering a better assessment of overfitting. The ROC-AUC scores across *k* = (3, 5, and 10) indicate robustness by a stable or slight variation as presented in Supporting Information (Tables S3, S4, and S5). Then we chose to perform most analysis using the 5-fold cross validation.

We would like to mention that ROC-AUC scores are recognized as good metrics for balanced data sets, however MCC and Kohen′s kappa are better metrics for imbalanced data sets. As our data sets are imbalanced, we preferred to use most of analysis using MCC and Kohen′s kappa metrics. Thus, we preferred to use the best metrics for imbalanced data sets.

The hyperparameter optimization of the RF classifier models obtained with the five subdata sets was investigated to find the best parameters. The optimized hyperparameters are presented in Table 2. While the number of estimators for the RF classifier models ranged between 129 and 446 for each run, the depth of the tree consistently remained 19. Using these optimum parameters, the ROC-AUC metrics for each classification model were always higher than 0.981 and 0.953 for the training and validation data sets, respectively, as shown in Table 3. This indicates an absence of substantial overfitting because 5-fold cross-validation was used.

**Table 2:** Optimized Parameters in the Hyperparameterization Process for Random Forest (RF) Classifier Models for the Endocrine Disruption of Compounds Using the EDC and EDKB-FDA Datasets^a^

Data set	Task	Depth	Estimators
EDC	AR	19	171
	ER	19	129
	AhR	19	280
	ARO	19	441
	PPAR	19	325
EDKB-FDA	AR	19	175
	ER	19	446
	AhR	19	315
	ARO	19	139
	PPAR	19	313

a AR stands for androgen receptors; ER—estrogen receptors; AhR—aryl hydrocarbon receptors; ARO—aromatase receptors; PPAR—peroxisome proliferator–activated receptors.

**Table 3:** Metrics (Mean ROC-AUC) of the Training and Validation Datasets for Random Forest (RF) Classifier Models for the Endocrine Disruption of Compounds Using the EDC and EDKB-FDA Datasets^a^

Data set	Task	Precision of Training	Precision of validation
EDC	AR	0.999	0.998
	ER	0.981	0.953
	AhR	0.996	0.990
	ARO	0.999	0.998
	PPAR	0.999	0.997
EDKB-FDA	AR	0.999	0.997
	ER	0.982	0.959
	AhR	0.997	0.989
	ARO	0.998	0.997
	PPAR	0.999	0.999

a AR stands for androgen receptors; ER—estrogen receptors; AhR—aryl hydrocarbon receptors; ARO—aromatase receptors; PPAR—peroxisome proliferator–activated receptors.

**Figure 1:**
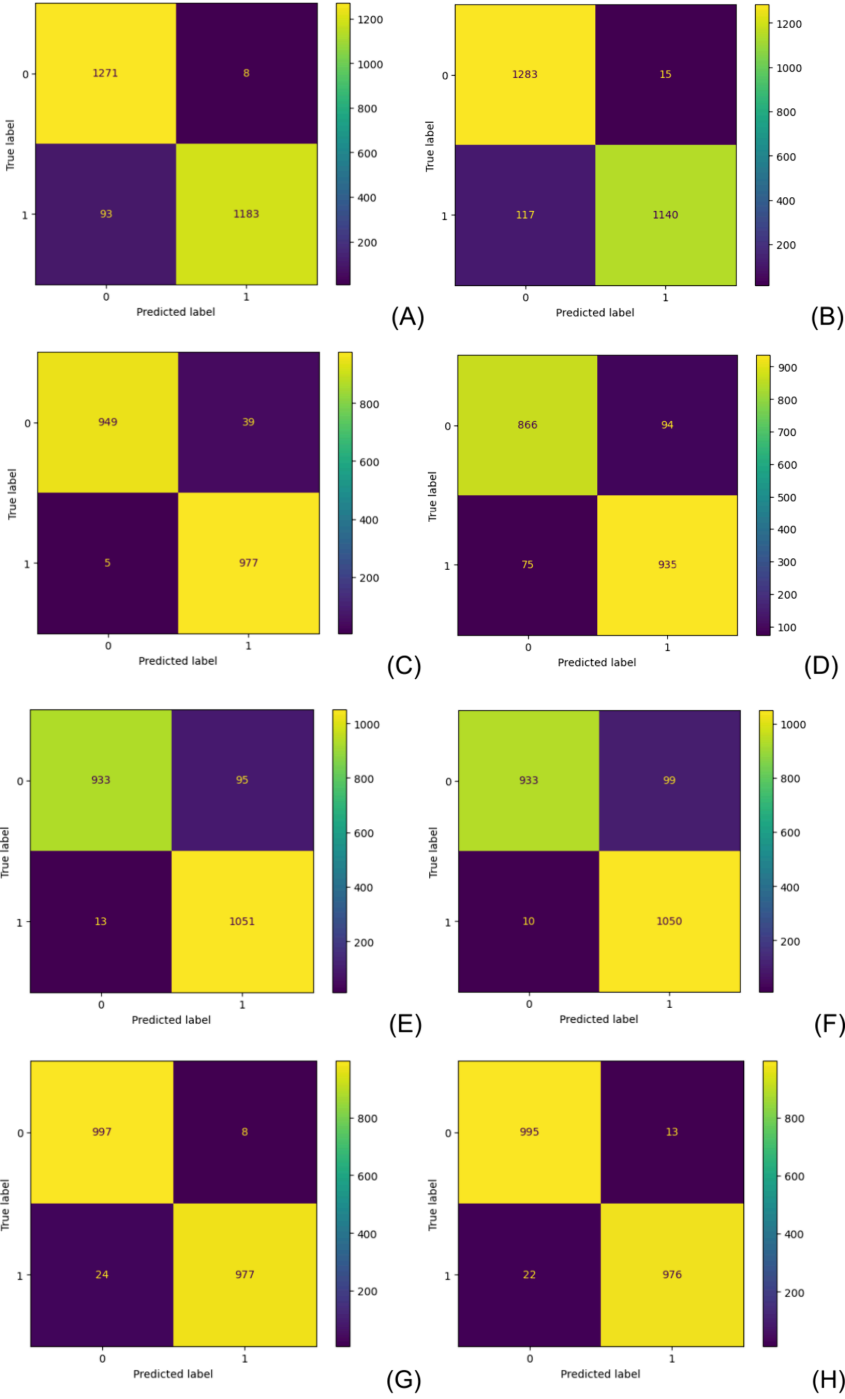
Confusion matrix for the RF classification model using the TOX-21 validation data set to build the model to be used for the EDC ((A) for AR, (C) for ER, (E) for AhR, (G) for ARO, and (I) for PPAR) and EDKB ((B) for AR, (D) for ER, (F) for AhR, (H) for ARO, and (J) for PPAR) test data sets.

The evaluation metrics, including precision, recall, F1 score, MCC and accuracy, used to assess the performance of classifier models in the context of endocrine disruptors are presented in Table 4. These metrics are described in terms of true positives (TP), false positives (FP), true negatives (TN), and false negatives (FN). The F1 score is the harmonic mean of precision and recall. The accuracy of the models was evaluated for both endocrine disruption and nonendocrine disruption, focusing particularly on the performance of RF classifier models. The confusion matrix, presented in Figure 1, was employed to probe the accuracy of classification for endocrine disruption. The importance of building separate models for endocrine disruption and nonendocrine disruption is underscored due to the aim of only obtaining substructures important to represent endocrine disruption. Both models perform well for endocrine disruption and nonendocrine disruption, yet our focus was solely on the models that describe endocrine disruption. Nevertheless, for this classification and for this data set, MCC is a more reliable score, because it only produces a high score if the prediction obtains good results in all four categories of the confusion matrix (TP, FN, TN, and FP), while proportionally considering the sizes of both positive and negative elements in the data set.

**Table 4:** Precision, Recall, F1, MCC and Accuracy Scores for Random Forest (RF) Classifier Models for the Validation Dataset of Endocrine Disruption Using the EDC and EDKB-FDA Datasets^a^

Data set	Task	Scores				
		Precision	Recall	F1	Accuracy	MCC
EDC	AR	0.993	0.927	0.960	0.960	0.923
	ER	0.962	0.995	0.977	0.977	0.956
	AhR	0.917	0.987	0.948	0.948	0.899
	ARO	0.991	0.976	0.984	0.984	0.968
	PPAR	0.989	0.973	0.982	0.982	0.964
EDKB-FDA	AR	0.987	0.907	0.948	0.948	0.899
	ER	0.909	0.926	0.914	0.914	0.828
	AhR	0.914	0.990	0.948	0.948	0.899
	ARO	0.987	0.978	0.983	0.983	0.965
	PPAR	0.993	0.989	0.991	0.991	0.982

a AR stands for androgen receptors; ER—estrogen receptors; AhR—aryl hydrocarbon receptors; ARO—aromatase receptors; PPAR—peroxisome proliferator–activated receptors.

The parameters of the LIME explainer instance were thoroughly investigated. The number of features was scrutinized. Although the classifier model incorporated 1024 features, less than a dozen were found to be significant for each sample. Adjusting this parameter had no discernible impact on the execution time or results. In our LIME code, it is appropriated to use 1024 fingerprints and used the 100 most important for each molecule as default.

We used a conservative setting of 100 features chosen for safety in the LIME interpretation. The impact of modifying the number of explanations on the probability of prediction for each substructure was also examined, varying from zero to 10, with higher values leading to more refined explanations, but with fewer quantity due to increased complexity. However, a reduction in the number of substructures with a weight greater than 0.1 was observed to be associated with higher values. Consequently, the number of explanations was set to 1, maximizing the quantity of meaningful substructures. It is important to note that higher values resulted in increased evaluation times, exceeding 4 h. This emphasizes the need to avoid excessively high values to ensure reasonable processing times during the evaluation process.

### Model Explanation

The LIME explanations for endocrine disruption are presented in the following. We filtered the results with weights greater than 0.1 to exclude less significant substructures. Then, LIME selected the most influential substructures for endocrine disruption for each one of the five nuclear receptors using the two testing data sets EDC and EDKB-FDA. Table S6 shows the summary of the most important chemical compounds classified by the classification models for the AR, ER, AhR, ARO, and PPAR targets. The full list of references related to the endocrine disruption of each chemical compound are in Table S6, not in the text below. The application of each chemical compound is also presented in Table S6. The structural alerts generated by our models are compared with the structural alerts found in literature for AR and ER acitivities.^4,20−23^ It is noteworthy to mention that most structural alerts generated here are more specific than those found in literature,^4,20−23^ i.e, most alerts are a portion of the structural alert (marked as P in Table S6) found in literature. Some structural alerts are found at vicinity (indicated as V in Table S6) of the alert found in literature. In these cases, it is possible to consider them as new structural alerts (specified as N in Table S6) because they are generally formed as different groups. In some situations (steroidal compounds and Forskolin), several structural alerts encompass most of the molecule structure. In summary, more specific structural alerts than existing ones are compared with those alerts found in the online chemical modeling (OCHEM) platform and/or in literature.^4,20−23^ Therefore, it is not possible to perform the statistical comparison of identified groups with set of already existing alerts. In terms of rigor and strictness, they have structural differences that prevent statistical confirmation of agreement. In fact, the structural alerts found here are mostly like those in literature.^4,20−23^ Unfortunately, it seems that each new explanation generated by different explainable artificial intelligence methods yields to different structural alerts depending on the model and data set used to train the model, but most alerts are a portion of the existing structural alert. On the other hand, as presented below, each new explanation brings some new knowledge, and in some degree, they are similar and comparable to previous studies.^4,20−23^

**Figure 2:**
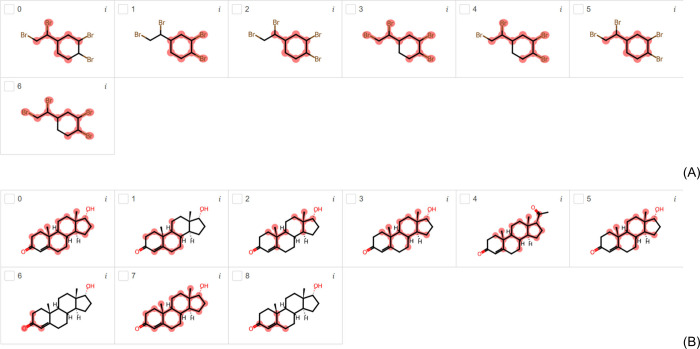
Most important substructures found by LIME for EDC (A) and EDKB-FDA (B) test data sets using the AR-EDC and AR-EDKB classification models.

Figures 2A and 2B present the endocrine disruptors highlighted using the AR model trained with the TOX21 and tested with the EDC and EDKB-FDA data sets. For the AR nuclear receptor, the 1,2-dibromo-4-(1,2-dibromoethyl)cyclohexane compound is the only one highlighted for endocrine disruption using the EDC data set in Figure 2A. It is used primarily as an additive flame retardant in polystyrene foams and contains α- and β- diastereoisomers present in equimolar amounts. The detection of this compound in the environment suggested that isomers are androgenic, indicating its potential risk to the environment due to toxic and biochemical effects of the β-isomer in a controlled laboratory environment.^26^ It is an agonist to human AR. In vivo, in vitro ligand-binding and receptor-activation assays demonstrated that this compound activates and binds to the human AR, indicating that it is a potential endocrine disruptor.^27^ There is preliminary evidence of disruption in endocrine and reproductive systems of birds and fish. Molecular docking, in vitro ligand-binding and receptor-activation assays present that 1,2-dibromo-4-(1,2-dibromoethyl)cyclohexane binds to and activates the human AR.^28^

Using the data set EDKB-FDA, Figure 2B shows the highlights of epitestosterone (compounds 0, 1, 2, 3, 5, 6, 7, and 8), which is an endogenous steroid and an epimer of the androgen sex hormone testosterone. It is a competitive AR antagonist and a masking agent for testosterone banned by many sporting authorities.^29^Figure 2B also presents the highlights of progesterone (compound 4), which stimulates the growth of mammary alveolar tissue and relaxes uterine smooth muscle. It binds to the progesterone and estrogen receptors, but it also has androgenic activity.^30^

**Figure 3:**
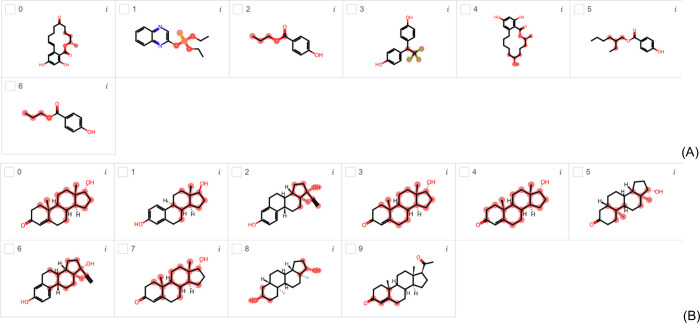
Most important substructures found by LIME for EDC (A) and EDKB-FDA (B) test data sets using the ER-EDC and ER-EDKB classification models.

Figures 3A and 3B show the endocrine disruptors highlighted using the ER model trained with the TOX21 and tested with the EDC and EDKB-FDA data sets. Using the EDC data set, zearalenone (compounds 0 and 4) is presented as ER endocrine disruptor in Figure 3A; however, it is a macrolide comprising a 14-membered lactone fused to the potent estrogenic metabolite, 1,3-dihydroxybenzene. It is a nonsteroidal compound with estrogenic activity. As this compound itself is not an endocrine disruptor, but its metabolite is, the highlights may indicate a misinterpretation of the real explanation for endocrine disruption. Therefore, it is important for the reader to pay attention to the LIME interpretation as it may lead to a wrong direction. However, the literature presents in vitro and in vivo evidence for the estrogen effects of the zearalenone and some of its metabolites.^31,32^ Compound 1 in Figure 3A is named as quinalphos, an organic thiophosphate compound used as a pesticide, which acts via acetylcholinesterase inhibition. Its thiophosphate group, like an ant antenna, is highlighted to be the cause of endocrine disruption and its shape is like several known endocrine disruptors. The link between endocrine disruptors and acetylcholinesterase (AC) is reported in literature.^33^ It decreases testosterone level considerably, and consequently results in reduced sperms in mice.^34^ Furthermore, it presents AhR-mediated transcriptional activity using in vitro assay.^35^ Compounds 2 (and 6) and 5 in Figure 3A are very similar. Propylparaben (compounds 2 and 6) and 2-Ethylhexyl 4-hydroxybenzoate (compound 5) are benzoate esters (parabens) that are known as endocrine disruptors. They are members of phenols and parabens, typically found in food additives, drug preservatives, and many cosmetics, such as lotions, shampoos, creams and bath products. They are also antifungal and antimicrobial agents. Their capacity to disrupt endocrine function via estrogen based on in vitro and in vivo data is presented in literature.^36,37^ Instead of highlighting the phenol group, LIME highlighted the ester group. Compound 3 in Figure 3A is a bisphenol A compound called hydroxychlor, a biologically active liver metabolite of methoxychlor that is commonly used as pesticide. It acts as an estrogen receptor agonist, and an antagonist at the estrogen receptor beta and androgen receptor.^38,39^ It may also disrupt the activities of aromatase.^40^ Instead of highlighting the phenol groups, LIME highlighted the trichloroethane group.

Figure 3B shows the highlights generated for the ER model using the EDKB-FDA data set. The compounds highlighted are epitestosterone (compounds 0, 3, 4, and 7), estradiol (compound 1), ethinyl estradiol (compounds 2 and 6), dihydrotestosterone (compound 5), 5α-androstane-3α,17β-diol (compound 8), and progesterone (compound 9). In fact, the concentrations of epitestosterone in human serum are negatively correlated with that of estradiol. The possible explanation for this relation and the influence of epitestosterone on estradiol formation in vitro are due to their structural similarity. As estradiol is an estrogen, the mechanism of action of estradiol is as an ER agonist like epitestosterone.^41^ Ethinyl estradiol is an estrogen synthesized from estradiol that exhibits high estrogenic disruption.^41^ Dihydrotestosterone is a testosterone in which the 4,5 double bond has been reduced to a single bond with alpha-configuration at position 5. It has a role as an androgen^42^ but it also inhibits estrogen accumulation in granulosa cells,^43,44^ and here it is highlighted by LIME as an estrogen-like molecule. 5α-androstane-3α,17β-diol is highlighted like the androgen metabolite, 5α-androstane-3β,17β-diol, that is a potent modulator of estrogen receptor.^45,46^ Progesterone is a C21-steroid hormone with a pregnane skeleton that attenuates beneficial neural effects of estrogen, and reduces ER-dependent transcriptional activity and neuroprotection by decreasing expression of ERα and ERβ receptors,^47^ as it is highlighted by LIME. Usually, LIME does not suggest any specific group in steroid-like molecules.

**Figure 4:**
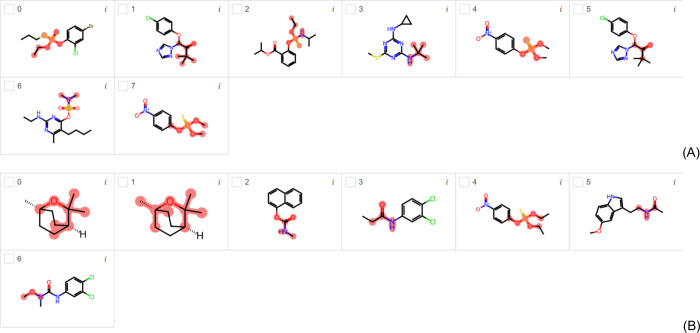
Most important substructures found by LIME for EDC (A) and EDKB-FDA (B) test data sets using the AhR-EDC and AhR-EDKB classification models.

Figures 4A and 4B present the endocrine disruptors highlighted using the AhR model trained with the TOX21 data set and validated with the EDC and EDKB-FDA data sets. Using the EDC data set, the compounds profenofos (compound 0), triadimefon (compounds 1 and 5), isofenphos (compound 2), cybutryne (compound 3), methyl parathion (compounds 4 and 7), and bupirimate (compound 6) are highlighted for AhR activity. Profenofos is an organophosphate insecticide, and member of monochlorobenzenes. It causes endocrine disruption and shows drastic effects on the testicular tissues in rabbits via AR and AhR activities.^35^ Triadimefon is a member of triazoles and monochlorobenzenes. It increases expression and enzymatic activities of a series of related cytochrome P450s (CYP), which is evidence to its effects on nuclear receptors, and causes thyroid endocrine disruption, but it is unknown to present AhR activity.^35^ Instead of the triazole and monochlorobenzene, the ketone group was found to be the cause of endocrine disruption by LIME. Isofenphos is an organothiophosphate insecticide that has a role as an AC inhibitor, and consequently, there is an association with endocrine disruption, but it does not present any AhR activity.^48^ The thiophosphate group in Profenofos and Isofenphos was found to be the cause of endocrine disruption by LIME. Cybutryne is a diamino-1,3,5-triazine that has a role as an organothiophosphate insecticide. No information regarding endocrine disruption was found for Cybutryne in literature. Instead of the triazine group, LIME found that the secondary amine group is the cause of endocrine disruption. Methyl parathion is an AC inhibitor; thus, its action mechanism of endocrine disruption is via AR activity.^48^ The thiophosphate group in Methyl Parathion was found to be the explanation for its endocrine disruption. Bupirimate is an antifungal agrochemical that has a role as an androgen antagonist. It also activates the pregnane X cellular receptor, but it is unknown to show AhR activity.^35,48^ The sulfamate ester group is highlighted to explain the cause of endocrine disruption in Bupirimate.

Using the EDKB-FDA data set (Figure 4B), eucalyptol (compounds 0 and 1), carbaryl (compound 2), propanil (compound 3), parathion (compound 4), melatonin (compound 5), and linuron (compound 6) are highlighted for AhR activity in Figure 4B. Eucalyptol is a monoterpenoid that controls airway mucus hypersecretion and asthma via anti-inflammatory cytokine inhibition; however it also has AR and ER activities, and it directly binds to AhR.^49^ The cyclic ether group was found to be the cause of endocrine disruption by LIME. Carbaryl is a carbamate ester that has a role as an AC inhibitor.^50^ It can activate AhR, and was first believed not to be an AhR ligand, but then it was proved to be a weak ligand.^51^ Later, it was also found to act as an AhR agonist in AhR-mediated reporter gene assays. Propanil is an anilide that has a role as herbicide with AC activity, and consequently, AR activity.^33^ It also has ER activity and a potent AhR agonist activity.^35,48^ Parathion is an organothiophosphate insecticide that has a role as an AC inhibitor, and consequently it has AR activity.^33^ It also activates AhR and stimulates the formation of an AhR-DNA-binding complex.^35,48^ Melatonin is a hormone secreted by the pineal gland in humans that has a role in the treatment of sleep disorders. Melatonin and its indolic and kynuric metabolites act as agonists to AhR, and are proved to stimulate the AhR protein translocation from the cytoplasm to the nucleus in human keratinocytes.^52^ It also has AR and ER activities.^53,54^ Linuron is a *N*-methyl phenylurea that has a role as herbicide. It is an AC inhibitor, and has AR and AhR activity.^35,48^ LIME found that the anilide group is the cause of endocrine disruption in Carbaryl, Propanil, Melatonin, and Linuron. On the other hand, the thiophosphate group in Parathion was found to be the explanation for its endocrine disruption.

**Figure 5:**
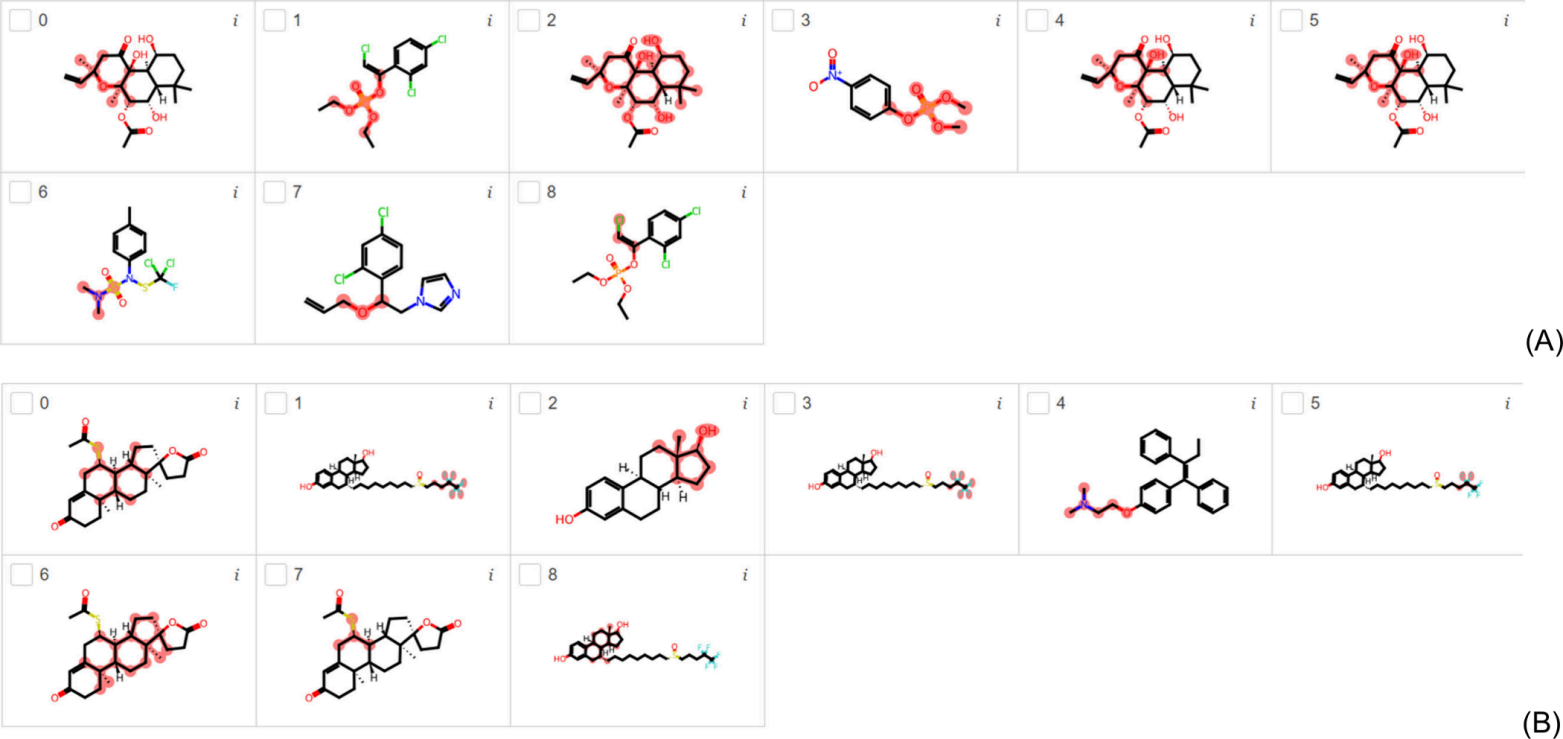
Most important substructures found by LIME for EDC (A) and EDKB-FDA (B) test data sets using the ARO-EDC and ARO-EDKB classification models.

Figures 5A and 5B show the endocrine disruptors highlighted using the ARO model trained with the TOX21 data set and tested with the EDC and EDKB-FDA data sets. Using the EDC data set, the compounds forskolin (compounds 0, 2, 4 and 5), chlorfenvinfos (compounds 1 and 8), methyl paraoxon (compound 3), tolylfluanid (compound 6), and enilconazole (compound 7) are highlighted for ARO activity in Figure 5A. Forskolin is a diterpenoid that has AR,^55^ ER,^56^ and ARO^57^ activities. It also produces PPAR isotype-specific mRNA, protein effects and a consistent decrease of ER-α mRNA levels.^58^ It was not found any specific group in Forskolin that causes the endocrine disruption. Chlorfenvinfos is a dichlorobenzene phosphate that has a role as an AC inhibitor.^35,48^ LIME found that the side-chain phosphorus group is the cause of endocrine disruption. Methyl paraoxon is the active metabolite of the organophosphate insecticide parathion that acts as a cholinesterase inhibitor.^35,48^ Paraoxon is one of the most potent acetylcholinesterase-inhibiting insecticides. The side-chain phosphorus group is found to be the cause of endocrine disruption by LIME. Tolylfluanid is a sulfamide that has a role as fungicide and wood preservative. The sulfamide group is highlighted by LIME to be the cause of endocrine disruption. To the best of our knowledge, the literature reports that it only has activity on the insulin receptor.^59^ It is identified as a potent endocrine disruptor using in vitro and ex vivo studies.^60^ Enilconazole is an imidazole that has role as fungicide with AR, ER, and ARO activities.^61^ It is a surprise that neither imidazole nor dichlorobenzene groups are the cause of endocrine disruption. Instead, the ether branched group was found by LIME to be the cause of endocrine disruption. The literature shows a need for in vivo studies to establish the extent of endocrine-disrupting effects.^62^

Using the EDKB-FDA data set (Figure 5B), the compounds S-fulvestrant (compound 1, 3, 5, and 8), estradiol (compound 2), and tamoxifen (compound 4) are highlighted for ARO activity in Figure 5B. Fulvestrant (S enantiomer) is an ER antagonist with a novel pharmacological profile, and it does not present agonist activity. It has been shown to significantly reduce cellular levels of the AR, ER and progesterone receptor in both preclinical studies and clinical trials of postmenopausal women with primary breast cancer.^63,64^ In addition, it exists information on its ARO activity that catalyzes estradiol synthesis.^65^ Estradiol can act as an antiaromatase agent. However, the factors regulating aromatase activity are unknown.^66^ Tamoxifen is a stilbenoid that has AR activity, is an ER antagonist and upregulates ARO expression.^67−69^

**Figure 6:**
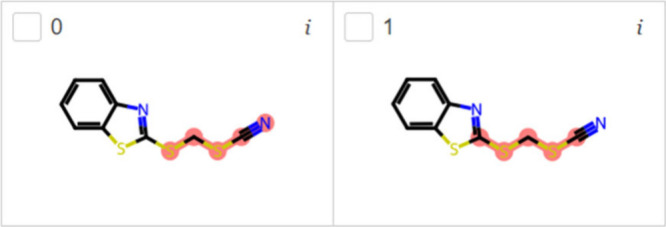
Most important substructures found by LIME for EDC (A) and EDKB-FDA (B) test data sets using the PPAR-EDC and PPAR-EDKB classification models.

Figure 6 shows the endocrine disruptor from the EDC data set highlighted using the PPAR model trained with the TOX21 data set and validated with the EDC and EDKB-FDA data sets. This model was not able to find any endocrine disruptor from the EDKB-FDA data set. The compound 2-(thiocyanomethylthio)benzothiazole from the EDC data set is a wood preservative, marine biocide, fungicide, and a preservative in paint. It is an emerging contaminant with only one study on PPAR activity and no activity for any other nuclear receptor activity up to date.^70^ LIME found that the side chain and thiocyanate group are the explanation for endocrine disruption in the 2-(thiocyanomethylthio)benzothiazole.

These structural explanations should not be regarded as strict rules. Our goal is to create structural interpretability using LIME and assess whether the most important toxic alerts for endocrine disruption align with human scientific understanding. It is important to acknowledge that LIME can make incorrect interpretations, which are not the reason for endocrine disruption. Although LIME highlights the side group in propylparaben (compounds 2 and 6 in Figure 3A), it is known as an inactive group, and the active group is the phenol group. Similarly, LIME seems to be nonspecific in some cases such as flame-retardant compounds (Figure 2A) and steroid-like compounds (Figure 2B). In both cases, LIME is unable to select one specific group and highlights the whole molecule. Another issue is evident in metabolites because the parent molecule is not the real endocrine disruptor, but LIME predicts the existing substructures in both the parent molecule and metabolite, and sometimes this substructure is not the reason for endocrine disruption as exemplified in zearalenone (compounds 0 and 4 in Figure 3A). In this case, the macrolide comprising a 14-membered lactone is not the cause of endocrine disruption, but its metabolite, 1,3-dihydroxybenzene, is. Despite these limitations, the interpretation of important substructures for endocrine disruption with a certain level of confidence can be used as a basis for analyzing counterfactuals, which suggest structural changes in molecules to reduce or eliminate their endocrine disruption.

## Concluding Remarks

The skepticism about the effectiveness of LIME in aiding researchers stems from several factors. The design of new molecules with desired safety in terms of toxicology is a complex problem. LIME can explain individual model predictions, but it may not fully capture the nuances of molecular interactions and structural factors for endocrine disruption. Its explanations are limited to instance-specific, local insights and may not provide a holistic view, which is essential for molecule design. Furthermore, the interpretation of results requires expertise in both chemistry and machine learning, making it challenging for researchers lacking strong backgrounds in both fields to use effectively. Any insights from LIME would still need experimental validation, which can be costly and time-consuming. Most importantly, LIME can offer insights into machine learning model predictions in toxicology, but it may have limited utility in guiding the synthesis of new molecules due to the lack of experimental findings about structural alerts. Researchers should consider these potential drawbacks and limitations when incorporating LIME into their workflow.

The use of state-of-the-art techniques in explainable machine learning applied to toxicology may limit the understanding and interpretation of predictions from complex machine learning models. LIME offers several advantages over traditional explainable machine learning methods. It provides instance-specific and localized explanations, which are often more intuitive and easier to understand than global model level explanations. It is model agnostic, i.e., it can be applied to any machine learning model, independent of complexity or underlying architecture. It also generates typically simpler, quicker, and more transparent explanations than other methods, enhancing accessibility for nonexperts and fostering trust in model predictions. The identification of important features that influence individual predictions allows researchers to discern the features driving the model predictions. Moreover, it sheds light into complex models, such as deep neural networks, where traditional explainable machine learning methods may struggle. Then, it can improve the ability of researchers to interpret and trust machine learning model predictions in toxicology, leading to more informed decision-making in environmental risk assessment, and environmental policy and regulation.

Some machine learning models treat the prediction task as a black box model, take the input derived from a chemical structure, and produce a predicted value. However, these models do not provide an explanation for their predictions by itself, making it difficult for the user to evaluate ideas. On the other hand, it would be valuable to have random forest models that provide human-interpretable explanations, driving subsequent molecule design.

Although there has not been much progress in explainable machine learning for molecules, toxicity data sets have been successfully interpreted using LIME.^14^ The challenge has been to get experimental evidence to validate the explanations of unknown compounds or their action molecular mechanisms in real biological entities. The explanation depends on the data set. Models built with a data set give different results from models created by another data set. Remarkably, it is important to focus benchmarking efforts on simple, robust, relevant, diverse, and clearly defined end points for endocrine disruption with different nuclear receptors. These end points must be reproducible, relatively inexpensive, and can provide reasonably large data sets, preferentially performed with the same methodology and in the same lab. Consequently, it would be possible to make better structural explanations for each nuclear receptor. Additionally, LIME^14^ may sometimes ignore substructures, which are normally responsible for endocrine disruption. Also, compounds with two substructures responsible for endocrine disruption are highlighted with the explanation of the most relevant one. Finally, it is also noteworthy to comment that these structural explanations cannot be interpreted as definitive rules. Nowadays, we focus on creating reliable structural interpretability from LIME18^15^ and assessing whether the most important substructures to endocrine disruption are consistent with existing human scientific understanding. And, in a future perspective, we can possibly predict new trustful molecular insights on each new endocrine disruptor using the developed models with benchmarking data sets.

The analysis of endocrine disruption using LIME explanations for the EDC and EDKB-FDA data sets reveals multiple significant substructures associated with the endocrine disruption of chemical compounds. They were classified in terms of interactions with five specific nuclear receptors: AR, ER, AhR, ARO, and PPAR. Notably, LIME identified substructures such as thiophosphate, sulfamate ester, anilide, carbamate, sulfamide, and thiocyanate groups as influential in endocrine disruption. It is important to acknowledge that some compounds presented only endocrine disruption data in animals, with no evidence in humans available in the literature. However, the comprehensive evaluation revealed instances where detected toxic alerts aligned with expectations and highlighted potential features associated with endocrine disruption. The relevance of LIME in explaining endocrine disruption is consistent across most outputs. In summary, the use of LIME to explain endocrine disruption in the EDC and EDKB-FDA data sets provides valuable insights into the specific substructures that contribute to the classification of compounds as endocrine disruptors. The detailed analysis of LIME results enhances the overall understanding of the endocrine disruption of the analyzed compounds that lack specific information in literature. Identifying of more specific toxic alerts that contribute to endocrine disruption is crucial for the development of alternative methods to evaluate the risks of chemical exposures, both on environmental and human health, without relying on animal testing. The outcomes of our findings go beyond theoretical scopes, providing tangible benefits for environmental monitoring agencies, regulatory agencies, and pharmaceutical and food industries. The capacity to accurately predict endocrine disruption grants decision-makers essential tools to assess the safety of chemicals and enhance environmental risk assessments.

## Data Availability

Code, input data and processing scripts for this paper are available at GitHub repository (https://github.com/andresilvapimentel/endocrine-disruption-explainer). The data analysis scripts of this paper are also available in the interactive notebook Google Colab.

## Supporting Information Available

The ROC-AUC, precision, recall, F1, accuracy, MCC and Cohen′s Kappa scores for Extra Trees (ET), Random Forest (RF), and Gaussian Naïve Bayes (GNB) classifier models for the train and validation data sets of endocrine disruption found in TOX21 data set. The external validation using the precision score for Random Forest (RF) classifier models in the EDC and EDKB-FDA data sets of endocrine disruption. The ROC-AUC, precision, recall, F1, accuracy, MCC, and Cohen′ Kappa scores for Random Forest (RF) classifier models for the train and validation data sets of endocrine disruption found in TOX21 data set using the (3, 5, and 10)-fold cross validation method. The full list of references related to the endocrine disruption of each chemical compound (PDF)

## References

[ref1] La Merrill M. A.; Vandenberg L. N.; Smith M. T.; Goodson W.; Browne P.; Patisaul H. B.; Guyton K. Z.; Kortenkamp A.; Cogliano V. J.; Woodruff T. J.; Rieswijk L.; Sone H.; Korach K. S.; Gore A. C.; Zeise L.; Zoeller R. T. Consensus on the Key Characteristics of Endocrine-Disrupting Chemicals as a Basis for Hazard Identification. Nat. Rev. Endocrinol 2020, 16 (1), 45–57. 10.1038/s41574-019-0273-8. 31719706 PMC6902641

[ref2] Chen Y.; Yang J.; Yao B.; Zhi D.; Luo L.; Zhou Y. Endocrine Disrupting Chemicals in the Environment: Environmental Sources, Biological Effects, Remediation Techniques, and Perspective. Environ. Pollut. 2022, 310, 119918 10.1016/j.envpol.2022.119918. 35952990

[ref3] Mukherjee A.; Su A.; Rajan K. Deep Learning Model for Identifying Critical Structural Motifs in Potential Endocrine Disruptors. J. Chem. Inf Model 2021, 61 (5), 2187–2197. 10.1021/acs.jcim.0c01409. 33872000

[ref4] Zhang R.; Wang B.; Li L.; Li S.; Guo H.; Zhang P.; Hua Y.; Cui X.; Li Y.; Mu Y.; Huang X.; Li X. Modeling and Insights into the Structural Characteristics of Endocrine-Disrupting Chemicals. Ecotoxicol Environ. Saf 2023, 263, 115251–115251. 10.1016/j.ecoenv.2023.115251. 37451095

[ref5] Jia X.; Wang T.; Zhu H. Advancing Computational Toxicology by Interpretable Machine Learning. Environ. Sci. Technol. 2023, 57 (46), 17690–17706. 10.1021/acs.est.3c00653. 37224004 PMC10666545

[ref6] Zhang J.; Norinder U.; Svensson F. Deep Learning-Based Conformal Prediction of Toxicity. J. Chem. Inf Model 2021, 61 (6), 2648–2657. 10.1021/acs.jcim.1c00208. 34043352

[ref7] Zorn K. M.; Foil D. H.; Lane T. R.; Hillwalker W.; Feifarek D. J.; Jones F.; Klaren W. D.; Brinkman A. M.; Ekins S. Comparison of Machine Learning Models for the Androgen Receptor. Environ. Sci. Technol. 2020, 54 (21), 13690–13700. 10.1021/acs.est.0c03984. 33085465 PMC8243727

[ref8] Sun L.; Yang H.; Cai Y.; Li W.; Liu G.; Tang Y. In Silico Prediction of Endocrine Disrupting Chemicals Using Single-Label and Multilabel Models. J. Chem. Inf Model 2019, 59 (3), 973–982. 10.1021/acs.jcim.8b00551. 30807141

[ref9] Sapounidou M.; Norinder U.; Andersson P. L. Predicting Endocrine Disruption Using Conformal Prediction - A Prioritization Strategy to Identify Hazardous Chemicals with Confidence. Chem. Res. Toxicol. 2023, 36 (1), 53–65. 10.1021/acs.chemrestox.2c00267. 36534483 PMC9846826

[ref10] Zorn K. M.; Foil D. H.; Lane T. R.; Russo D. P.; Hillwalker W.; Feifarek D. J.; Jones F.; Klaren W. D.; Brinkman A. M.; Ekins S. Machine Learning Models for Estrogen Receptor Bioactivity and Endocrine Disruption Prediction. Environ. Sci. Technol. 2020, 54 (19), 12202–12213. 10.1021/acs.est.0c03982. 32857505 PMC8194504

[ref11] Yu Z.; Wu Z.; Zhou M.; Cao K.; Li W.; Liu G.; Tang Y. EDC-Predictor: A Novel Strategy for Prediction of Endocrine-Disrupting Chemicals by Integrating Pharmacological and Toxicological Profiles. Environ. Sci. Technol. 2023, 57 (46), 18013–18025. 10.1021/acs.est.2c08558. 37053516

[ref12] Halder A. K.; Moura A. S.; Cordeiro M. N. D. S. Predicting the Ecotoxicity of Endocrine Disruptive Chemicals: Multitasking in Silico Approaches towards Global Models. Sci. Total Environ. 2023, 889, 164337 10.1016/j.scitotenv.2023.164337. 37211130

[ref13] Gou Y.; Shen L.; Cui S.; Huang M.; Wu Y.; Li P.; Zhuang S. Machine Learning Based Models for High-Throughput Classification of Human Pregnane X Receptor Activators. Environ. Sci.: Adv. 2023, 2 (2), 304–312. 10.1039/D2VA00182A.

[ref14] Nascimento C. M. C.; Moura P. G.; Pimentel A. S. Generating Structural Alerts from Toxicology Datasets Using the Local Interpretable Model-Agnostic Explanations Method. Digit Discovery 2023, 2 (5), 1311–1325. 10.1039/D2DD00136E.

[ref15] Ribeiro M. T.; Singh S.; Guestrin C. Why Should I Trust You?”: Explaining the Predictions of Any Classifier. arXiv 2016, 04938 10.48550/arXiv.1602.04938.

[ref16] Tan H.; Chen Q.; Hong H.; Benfenati E.; Gini G. C.; Zhang X.; Yu H.; Shi W. Structures of Endocrine-Disrupting Chemicals Correlate with the Activation of 12 Classic Nuclear Receptors. Environ. Sci. Technol. 2021, 55 (24), 16552–16562. 10.1021/acs.est.1c04997. 34859678

[ref17] Richard A. M.; Huang R.; Waidyanatha S.; Shinn P.; Collins B. J.; Thillainadarajah I.; Grulke C. M.; Williams A. J.; Lougee R. R.; Judson R. S.; Houck K. A.; Shobair M.; Yang C.; Rathman J. F.; Yasgar A.; Fitzpatrick S. C.; Simeonov A.; Thomas R. S.; Crofton K. M.; Paules R. S.; Bucher J. R.; Austin C. P.; Kavlock R. J.; Tice R. R. The Tox21 10K Compound Library: Collaborative Chemistry Advancing Toxicology. Chem. Res. Toxicol. 2021, 34 (2), 189–216. 10.1021/acs.chemrestox.0c00264. 33140634 PMC7887805

[ref18] Montes-Grajales D.; Olivero-Verbel J. EDCs Databank: 3D-Structure Database of Endocrine Disrupting Chemicals. Toxicology 2015, 327, 87–94. 10.1016/j.tox.2014.11.006. 25451822

[ref19] Ding D.; Xu L.; Fang H.; Hong H.; Perkins R.; Harris S.; Bearden E. D.; Shi L.; Tong W. The EDKB: An Established Knowledge Base for Endocrine Disrupting Chemicals. BMC Bioinformatics 2010, 11 (S6), S5 10.1186/1471-2105-11-S6-S5. PMC3026379 20946616

[ref20] Nendza M.; Wenzel A.; Müller M.; Lewin G.; Simetska N.; Stock F.; Arning J. Screening for Potential Endocrine Disruptors in Fish: Evidence from Structural Alerts and in Vitro and in Vivo Toxicological Assays. Environ. Sci. Eur. 2016, 28 (1), 26 10.1186/s12302-016-0094-5. 27867807 PMC5093190

[ref21] Fang H.; Tong W.; Branham W. S.; Moland C. L.; Dial S. L.; Hong H.; Xie Q.; Perkins R.; Owens W.; Sheehan D. M. Study of 202 Natural, Synthetic, and Environmental Chemicals for Binding to the Androgen Receptor. Chem. Res. Toxicol. 2003, 16 (10), 1338–1358. 10.1021/tx030011g. 14565775

[ref22] Hong H.; Tong W.; Fang H.; Shi L.; Xie Q.; Wu J.; Perkins R.; Walker J. D.; Branham W.; Sheehan D. M. Prediction of Estrogen Receptor Binding for 58,000 Chemicals Using an Integrated System of a Tree-Based Model with Structural Alerts. Environ. Health Perspect 2002, 110 (1), 29–36. 10.1289/ehp.0211029. 11781162 PMC1240690

[ref23] Blair R. M. The Estrogen Receptor Relative Binding Affinities of 188 Natural and Xenochemicals: Structural Diversity of Ligands. Toxicol. Sci. 2000, 54 (1), 138–153. 10.1093/toxsci/54.1.138. 10746941

[ref24] Rosa L. C. S.; Pimentel A. S. Applying Local Interpretable Model-Agnostic Explanations to Identify Substructures That Are Responsible for Mutagenicity of Chemical Compounds. Mol. Syst. Des Eng. 2024, 9 (9), 920–936. 10.1039/D4ME00038B.

[ref25] Rosa L. C. S.; Argolo C. O.; Nascimento C. M. C.; Pimentel A. S. Identifying Substructures That Facilitate Compounds to Penetrate the Blood–Brain Barrier via Passive Transport Using Machine Learning Explainer Models. ACS Chem. Neurosci. 2024, 15 (11), 2144–2159. 10.1021/acschemneuro.3c00840. 38723285 PMC11157485

[ref26] Ruan Y.; Lam J. C. W.; Zhang X.; Lam P. K. S. Temporal Changes and Stereoisomeric Compositions of 1,2,5,6,9,10-Hexabromocyclododecane and 1,2-Dibromo-4-(1,2-Dibromoethyl)Cyclohexane in Marine Mammals from the South China Sea. Environ. Sci. Technol. 2018, 52 (5), 2517–2526. 10.1021/acs.est.7b05387. 29397695

[ref27] Curran I. H. A.; Liston V.; Nunnikhoven A.; Caldwell D.; Scuby M. J. S.; Pantazopoulos P.; Rawn D. F. K.; Coady L.; Armstrong C.; Lefebvre D. E.; Bondy G. S. Toxicologic Effects of 28-Day Dietary Exposure to the Flame Retardant 1,2-Dibromo-4-(1,2-Dibromoethyl)-Cyclohexane (TBECH) in F344 Rats. Toxicology 2017, 377, 1–13. 10.1016/j.tox.2016.12.001. 27932249

[ref28] Pradhan A.; Asnake S.; Kharlyngdoh J. B.; Modig C.; Olsson P.-E. In Silico and Biological Analysis of Anti-Androgen Activity of the Brominated Flame Retardants ATE, BATE and DPTE in Zebrafish. Chem. Biol. Interact 2015, 233, 35–45. 10.1016/j.cbi.2015.03.023. 25818047

[ref29] Stárka L.; Bičíková M.; Hampl R. Epitestosterone—an Endogenous Antiandrogen?. J. Steroid Biochem 1989, 33 (5), 1019–1021. 10.1016/0022-4731(89)90255-0. 2532272

[ref30] O’Shaughnessy P. J.; Antignac J. P.; Le Bizec B.; Morvan M.-L.; Svechnikov K.; Söder O.; Savchuk I.; Monteiro A.; Soffientini U.; Johnston Z. C.; Bellingham M.; Hough D.; Walker N.; Filis P.; Fowler P. A. Alternative (Backdoor) Androgen Production and Masculinization in the Human Fetus. PLoS Biol. 2019, 17 (2), e3000002 10.1371/journal.pbio.3000002. 30763313 PMC6375548

[ref31] Chi M. S.; Mirocha C. J.; Weaver G. A.; Kurtz H. J. Effect of Zearalenone on Female White Leghorn Chickens. Appl. Environ. Microbiol. 1980, 39 (5), 1026–1030. 10.1128/aem.39.5.1026-1030.1980. 6446881 PMC291469

[ref32] Pompa G.; Montesissa C.; Di Lauro F. M.; Fadini L. The Metabolism of Zearalenone in Subcellular Fractions from Rabbit and Hen Hepatocytes and Its Estrogenic Activity in Rabbits. Toxicology 1986, 42 (1), 69–75. 10.1016/0300-483X(86)90093-4. 2948296

[ref33] Schug T. T.; Blawas A. M.; Gray K.; Heindel J. J.; Lawler C. P. Elucidating the Links Between Endocrine Disruptors and Neurodevelopment. Endocrinology 2015, 156 (6), 1941–1951. 10.1210/en.2014-1734. 25714811 PMC5393340

[ref34] Kokilavani P.; Suriyakalaa U.; Elumalai P.; Abirami B.; Ramachandran R.; Sankarganesh A.; Achiraman S. Antioxidant Mediated Ameliorative Steroidogenesis by Commelina Benghalensis L. and Cissus Quadrangularis L. against Quinalphos Induced Male Reproductive Toxicity. Pestic. Biochem. Physiol. 2014, 109, 18–33. 10.1016/j.pestbp.2014.01.002. 24581381

[ref35] Takeuchi S.; Iida M.; Yabushita H.; Matsuda T.; Kojima H. In Vitro Screening for Aryl Hydrocarbon Receptor Agonistic Activity in 200 Pesticides Using a Highly Sensitive Reporter Cell Line, DR-EcoScreen Cells, and in Vivo Mouse Liver Cytochrome P450–1A Induction by Propanil, Diuron and Linuron. Chemosphere 2008, 74 (1), 155–165. 10.1016/j.chemosphere.2008.08.015. 18835618

[ref36] Gazin V.; Marsden E.; Marguerite F. Oral Propylparaben Administration to Juvenile Male Wistar Rats Did Not Induce Toxicity in Reproductive Organs. Toxicol. Sci. 2013, 136 (2), 392–401. 10.1093/toxsci/kft211. 24068675

[ref37] Martín J. M. P.; Freire P. F.; Daimiel L.; Martínez-Botas J.; Sánchez C. M.; Lasunción M. Á.; Peropadre A.; Hazen M. J. The Antioxidant Butylated Hydroxyanisole Potentiates the Toxic Effects of Propylparaben in Cultured Mammalian Cells. Food Chem. Toxicol. 2014, 72, 195–203. 10.1016/j.fct.2014.07.031. 25086368

[ref38] Boberg J.; Taxvig C.; Christiansen S.; Hass U. Possible Endocrine Disrupting Effects of Parabens and Their Metabolites. Reprod. Toxicol. 2010, 30 (2), 301–312. 10.1016/j.reprotox.2010.03.011. 20381602

[ref39] Bolger R.; Wiese T. E.; Ervin K.; Nestich S.; Checovich W. Rapid Screening of Environmental Chemicals for Estrogen Receptor Binding Capacity. Environ. Health Perspect 1998, 106 (9), 551–557. 10.1289/ehp.98106551. 9721254 PMC1533147

[ref40] Liu S.; Mao B.; Bai Y.; Liu J.; Li H.; Li X.; Lian Q.; Ge R.-S. Effects of Methoxychlor and Its Metabolite Hydroxychlor on Human Placental 3β-Hydroxysteroid Dehydrogenase 1 and Aromatase in JEG-3 Cells. Pharmacology 2016, 97 (3–4), 126–133. 10.1159/000442711. 26735933

[ref41] Kuhl H. Pharmacology of Estrogens and Progestogens: Influence of Different Routes of Administration. Climacteric 2005, 8 (sup1), 3–63. 10.1080/13697130500148875. 16112947

[ref42] Grino P. B.; Griffin J. E.; Wilson J. D. Testosterone at High Concentrations Interacts with the Human Androgen Receptor Similarly to Dihydrotestosterone. Endocrinology 1990, 126 (2), 1165–1172. 10.1210/endo-126-2-1165. 2298157

[ref43] Conway B.-A.; Mills T. M. In Vitro Effects of Dihydrotestosterone on Granulosa Cell Production of Estrogen and Progesterone. Steroids 1991, 56 (5), 258–262. 10.1016/0039-128X(91)90044-V. 1877065

[ref44] Horton A. C.; Wilkinson M. M.; Kilanowski-Doroh I.; Dong Z.; Liu J.; Ogola B. O.; Visniauskas B.; Lindsey S. H. Dihydrotestosterone Induces Arterial Stiffening in Female Mice. Biol. Sex Differ 2024, 15 (1), 9 10.1186/s13293-024-00586-3. 38263051 PMC10804721

[ref45] Pak T. R.; Chung W. C. J.; Lund T. D.; Hinds L. R.; Clay C. M.; Handa R. J. The Androgen Metabolite, 5α-Androstane-3β, 17β-Diol, Is a Potent Modulator of Estrogen Receptor-B1-Mediated Gene Transcription in Neuronal Cells. Endocrinology 2005, 146 (1), 147–155. 10.1210/en.2004-0871. 15471969

[ref46] Handa R. J.; Weiser M. J.; Zuloaga D. G. A Role for the Androgen Metabolite, 5α-Androstane-3β,17β-Diol, in Modulating Oestrogen Receptor B-Mediated Regulation of Hormonal Stress Reactivity. J. Neuroendocrinol 2009, 21 (4), 351–358. 10.1111/j.1365-2826.2009.01840.x. 19207807 PMC2727750

[ref47] Jayaraman A.; Pike C. J. Progesterone Attenuates Oestrogen Neuroprotection Via Downregulation of Oestrogen Receptor Expression in Cultured Neurones. J. Neuroendocrinol 2009, 21 (1), 77–81. 10.1111/j.1365-2826.2008.01801.x. 19094096 PMC2692678

[ref48] Kojima H.; Takeuchi S.; Nagai T. Endocrine-Disrupting Potential of Pesticides via Nuclear Receptors and Aryl Hydrocarbon Receptor. J. Health Sci. 2010, 56 (4), 374–386. 10.1248/jhs.56.374.

[ref49] Ramsey J. T.; Li Y.; Arao Y.; Naidu A.; Coons L. A.; Diaz A.; Korach K. S. Lavender Products Associated With Premature Thelarche and Prepubertal Gynecomastia: Case Reports and Endocrine-Disrupting Chemical Activities. J. Clin Endocrinol Metab 2019, 104 (11), 5393–5405. 10.1210/jc.2018-01880. 31393563 PMC6773459

[ref50] Casado S.; Alonso M.; Herradón B.; Tarazona J. V.; Navas J. M. Activation of the Aryl Hydrocarbon Receptor by Carbaryl: Computational Evidence of the Ability of Carbaryl to Assume a Planar Conformation. Environ. Toxicol. Chem. 2006, 25 (12), 3141–3147. 10.1897/06-131R.1. 17220082

[ref51] Denison M. S.; Phelan D.; Winter G. M.; Ziccardi M. H. Carbaryl, a Carbamate Insecticide, Is a Ligand for the Hepatic Ah (Dioxin) Receptor. Toxicol. Appl. Pharmacol. 1998, 152 (2), 406–414. 10.1006/taap.1998.9999. 9853009

[ref52] Slominski A. T.; Kim T.-K.; Slominski R. M.; Song Y.; Qayyum S.; Placha W.; Janjetovic Z.; Kleszczyński K.; Atigadda V.; Song Y.; Raman C.; Elferink C. J.; Hobrath J. V.; Jetten A. M.; Reiter R. J. Melatonin and Its Metabolites Can Serve as Agonists on the Aryl Hydrocarbon Receptor and Peroxisome Proliferator-Activated Receptor Gamma. Int. J. Mol. Sci. 2023, 24 (20), 15496 10.3390/ijms242015496. 37895177 PMC10607054

[ref53] Rimler A.; Culig Z.; Lupowitz Z.; Zisapel N. Nuclear Exclusion of the Androgen Receptor by Melatonin. J. Steroid Biochem Mol. Biol. 2002, 81 (1), 77–84. 10.1016/S0960-0760(02)00050-X. 12127045

[ref54] Cos S.; Gonzalez A.; Martinez-Campa C.; Mediavilla M.; Alonso-Gonzalez C.; Sanchez-Barcelo E. Melatonin as a Selective Estrogen Enzyme Modulator. Curr. Cancer Drug Targets 2008, 8 (8), 691–702. 10.2174/156800908786733469. 19075592

[ref55] Blok L. J.; de Ruiter P. E.; Brinkmann A. O. Forskolin-Induced Dephosphorylation of the Androgen Receptor Impairs Ligand Binding. Biochemistry 1998, 37 (11), 3850–3857. 10.1021/bi9724422. 9521705

[ref56] Tsai H.-W.; Lin V. Y.; Shupnik M. A. Forskolin Stimulates Estrogen Receptor (ER) α Transcriptional Activity and Protects ER from Degradation by Distinct Mechanisms. Int. J. Endocrinol 2022, 2022, 1–17. 10.1155/2022/7690166. PMC9110234 35586275

[ref57] Sanderson J. T. Induction and Inhibition of Aromatase (CYP19) Activity by Natural and Synthetic Flavonoid Compounds in H295R Human Adrenocortical Carcinoma Cells. Toxicol. Sci. 2004, 82 (1), 70–79. 10.1093/toxsci/kfh257. 15319488

[ref58] Pavlikova N.; Kortner T. M.; Arukwe A. Peroxisome Proliferator-Activated Receptors, Estrogenic Responses and Biotransformation System in the Liver of Salmon Exposed to Tributyltin and Second Messenger Activator. Aquat. Toxicol. 2010, 99 (2), 176–185. 10.1016/j.aquatox.2010.04.014. 20466441

[ref59] Sargis R. M.; Neel B. A.; Brock C. O.; Lin Y.; Hickey A. T.; Carlton D. A.; Brady M. J. The Novel Endocrine Disruptor Tolylfluanid Impairs Insulin Signaling in Primary Rodent and Human Adipocytes through a Reduction in Insulin Receptor Substrate-1 Levels. Biochim. Biophys. Acta, Mol. Basis Dis. 2012, 1822 (6), 952–960. 10.1016/j.bbadis.2012.02.015. PMC3338892 22387882

[ref60] Regnier S. M.; Kirkley A. G.; Ye H.; El-Hashani E.; Zhang X.; Neel B. A.; Kamau W.; Thomas C. C.; Williams A. K.; Hayes E. T.; Massad N. L.; Johnson D. N.; Huang L.; Zhang C.; Sargis R. M. Dietary Exposure to the Endocrine Disruptor Tolylfluanid Promotes Global Metabolic Dysfunction in Male Mice. Endocrinology 2015, 156 (3), 896–910. 10.1210/en.2014-1668. 25535829 PMC4330315

[ref61] Jin C.; Zhang R.; Fu Z.; Jin Y. Maternal Exposure to Imazalil Disrupts the Endocrine System in F1 Generation Mice. Mol. Cell. Endocrinol. 2019, 486, 105–112. 10.1016/j.mce.2019.03.002. 30853599

[ref62] Kugathas S.; Audouze K.; Ermler S.; Orton F.; Rosivatz E.; Scholze M.; Kortenkamp A. Effects of Common Pesticides on Prostaglandin D2 (PGD2) Inhibition in SC5Mouse Sertoli Cells, Evidence of Binding at the COX-2 Active Site, and Implications for Endocrine Disruption. Environ. Health Perspect 2016, 124 (4), 452–459. 10.1289/ehp.1409544. 26359731 PMC4829986

[ref63] Lai A. C.; Crews C. M. Induced Protein Degradation: An Emerging Drug Discovery Paradigm. Nat. Rev. Drug Discov 2017, 16 (2), 101–114. 10.1038/nrd.2016.211. 27885283 PMC5684876

[ref64] Robertson J. F. R.; Harrison M. Fulvestrant: Pharmacokinetics and Pharmacology. Br. J. Cancer 2004, 90 (S1), S7–S10. 10.1038/sj.bjc.6601630. 15094758 PMC2750771

[ref65] Teodoro M. I.; Mayer A.; da Costa Miranda A.; Nunes H.; da Costa F. A.; Lourenço A. Real-World Effectiveness of Aromatase Inhibitors and Fulvestrant in HR+/HER2- Advanced Breast Cancer: A Snapshot of the Last Two Years before Conventional Use of CDK 4/6 Inhibitors in a Portuguese Institution. J. Pharm. Policy Pract 2024, 17 (1), 2296551 10.1080/20523211.2023.2296551. 38250517 PMC10798277

[ref66] Pasqualini J. R.; Chetrite G. S. Estradiol as an Anti-Aromatase Agent in Human Breast Cancer Cells. J. Steroid Biochem Mol. Biol. 2006, 98 (1), 12–17. 10.1016/j.jsbmb.2005.10.001. 16413774

[ref67] Viani G. A.; Bernardes da Silva L. G.; Stefano E. J. Prevention of Gynecomastia and Breast Pain Caused by Androgen Deprivation Therapy in Prostate Cancer: Tamoxifen or Radiotherapy?. Int. J. Radiat. Oncol. Biol. Phys. 2012, 83 (4), e519–e524. 10.1016/j.ijrobp.2012.01.036. 22704706

[ref68] Catalano S.; Giordano C.; Panza S.; Chemi F.; Bonofiglio D.; Lanzino M.; Rizza P.; Romeo F.; Fuqua S. A. W.; Maggiolini M.; Andò S.; Barone I. Tamoxifen through GPER Upregulates Aromatase Expression: A Novel Mechanism Sustaining Tamoxifen-Resistant Breast Cancer Cell Growth. Breast Cancer Res. Treat 2014, 146 (2), 273–285. 10.1007/s10549-014-3017-4. 24928526

[ref69] Loi S.; Criscitiello; Fumagalli; Saini Tamoxifen in Early-Stage Estrogen Receptor-Positive Breast Cancer: Overview of Clinical Use and Molecular Biomarkers for Patient Selection. Onco Targets Ther 2010, 4, 1–11. 10.2147/OTT.S10155. 21552410 PMC3084302

[ref70] Bertoldi C.; Pena A. C. C.; Dallegrave A.; Fernandes A. N.; Gutterres M. Photodegradation of Emerging Contaminant 2-(Tiocyanomethylthio) Benzothiazole (TCMTB) in Aqueous Solution: Kinetics and Transformation Products. Bull. Environ. Contam. Toxicol. 2020, 105 (3), 433–439. 10.1007/s00128-020-02954-2. 32740745

