# Supplementary material for: Toxic Alerts of Endocrine Disruption Revealed by Explainable Artificial Intelligence
Source: Environ Health (Wash). 2025 Jan 27;3(3):321–33. doi: 10.1021/envhealth.4c00218 (PMC11934200; doi:10.1021/envhealth.4c00218)
Supplement: Supplementary file 1 — eh4c00218_si_001.pdf [file eh4c00218_si_001.pdf]

Supporting Information

Toxic Alerts of Endocrine Disruption Revealed by Explainable Artificial Intelligence

Lucca Caiaffa Santos Rosa, Mariam Sarhan, and Andre Silva Pimentel*

Departamento de Química, Pontifícia Universidade Católica do Rio de Janeiro, Rio de Janeiro, RJ 22453-900, Brazil.

*Corresponding author: a_pimentel@puc-rio.br

Table S1. The ROC-AUC, precision, recall, F1, accuracy, MCC and Cohen´s Kappa scores for Extra Trees (ET), Random Forest (RF), and Gaussian Naïve Bayes (GNB) classifier models for the train and validation datasets of endocrine disruption found in TOX21 dataset. AR stands for androgen receptors, ER estrogen receptors, AhR aryl hydrocarbon receptors, ARO aromatase receptors, PPAR peroxisome proliferator–activated receptors.

**Train for AR**	**ROC AUC**	**Accuracy**	**Precision**	**Recall**	**F1**	**MCC**	**Kohen´ kappa**
**ET**	0.9999	0.9999	0.9998	1.0000	0.9999	0.9998	0.9998
**RF**	0.9999	0.9999	0.9998	1.0000	0.9999	0.9998	0.9998
**GNB**	0.7782	0.7782	0.8611	0.6635	0.7495	0.5717	0.5564
**Validation for AR**							
**RF**	0.9977	0.9977	0.9953	1.0000	0.9977	0.9953	0.9953
**Train for ER**	**ROC AUC**	**Accuracy**	**Precision**	**Recall**	**F1**	**MCC**	**Kohen´ kappa**
**ET**	0.9999	0.9999	0.9997	1.0000	0.9999	0.9997	0.9997
**RF**	0.9999	0.9999	0.9997	1.0000	0.9999	0.9997	0.9997
**GNB**	0.7109	0.7109	0.7714	0.5995	0.6747	0.4327	0.4218
**Validation for ER**							
**RF**	0.9909	0.9909	0.9821	1.0000	0.9909	0.9819	0.9817
**Train for AhR**	**ROC AUC**	**Accuracy**	**Precision**	**Recall**	**F1**	**MCC**	**Kohen´ kappa**
**ET**	0.9999	0.9999	0.9998	1.0000	0.9999	0.9998	0.9998
**RF**	0.9999	0.9999	0.9998	1.0000	0.9999	0.9998	0.9998
**GNB**	0.8264	0.8264	0.8275	0.8246	0.8261	0.6527	0.6527
**Validation for AhR**							
**RF**	0.9857	0.9857	0.9748	0.9971	0.9858	0.9716	0.9713
**Train for ARO**	**ROC AUC**	**Accuracy**	**Precision**	**Recall**	**F1**	**MCC**	**Kohen´ kappa**
**ET**	0.9999	0.9999	0.9998	1.0000	0.9999	0.9998	0.9998
**RF**	0.9999	0.9999	0.9998	1.0000	0.9999	0.9998	0.9998
**GNB**	0.7790	0.7790	0.7920	0.7567	0.7739	0.5585	0.5580
**Validation for ARO**							
**RF**	0.9990	0.9990	0.9980	1.0000	0.9990	0.9980	0.9980
**Train for PPAR**	**ROC AUC**	**Accuracy**	**Precision**	**Recall**	**F1**	**MCC**	**Kohen´ kappa**
**ET**	1.0000	1.0000	1.0000	1.0000	1.0000	1.0000	1.0000
**RF**	1.0000	1.0000	1.0000	1.0000	1.0000	1.0000	1.0000
**GNB**	0.7595	0.7596	0.6797	0.9819	0.8033	0.5796	0.5191
**Validation for PPAR**							
**RF**	0.9996	0.9996	0.9991	1.0000	0.9996	0.9991	0.9991

Table S2. The external validation using the precision score for Random Forest (RF) classifier models in the EDC and EDKB-FDA datasets of endocrine disruption. AR stands for androgen receptors, ER estrogen receptors, AhR aryl hydrocarbon receptors, ARO aromatase receptors, PPAR peroxisome proliferator–activated receptors. The number of EDC and EDKB-FDA compounds found in the external validation is precise.

Receptor	EDC compounds	Precision	EDKB-FDA compounds	Precision
**AR**	10	1.0	47	1.0
**ER**	188	1.0	138	1.0
**AhR**	211	1.0	35	1.0
**ARO**	61	1.0	14	1.0
**PPAR**	23	1.0	1	1.0

Table S3. The ROC-AUC, precision, recall, F1, accuracy, MCC and Cohen´ Kappa scores for Random Forest (RF) classifier models for the train and validation datasets of endocrine disruption found in TOX21 dataset using the 3-fold cross validation method. AR stands for androgen receptors, ER estrogen receptors, AhR aryl hydrocarbon receptors, ARO aromatase receptors, PPAR peroxisome proliferator–activated receptors.

**Task /Train**	**Scores**						
	**ROC-AUC**	**Precision**	**Recall**	**F1**	**Accuracy**	**MCC**	**Cohen' Kappa**
**AR**	0.9998	0.9995	0.9405	0.9700	0.9700	0.9417	0.9401
**ER**	0.9826	0.9583	0.9116	0.9360	0.9360	0.8731	0.8720
**AhR**	0.9970	0.9563	0.9938	0.9740	0.9740	0.9488	0.9481
**ARO**	0.9984	0.9979	0.9664	0.9821	0.9821	0.9648	0.9643
**PPAR**	0.9998	0.9975	0.9861	0.9919	0.9919	0.9839	0.9838
**Task /Validation**	**Scores**						
	**ROC-AUC**	**Precision**	**Recall**	**F1**	**Accuracy**	**MCC**	**Cohen´ Kappa**
**AR**	0.9981	0.9933	0.9408	0.9675	0.9675	0.9362	0.9350
**ER**	0.9629	0.9082	0.8935	0.90152	0.9015	0.8031	0.8031
**AhR**	0.9896	0.8908	0.9902	0.9359	0.9359	0.8773	0.8721
**ARO**	0.9957	0.9857	0.9698	0.9780	0.9780	0.9562	0.9561
**PPAR**	0.9991	0.9913	0.9777	0.9843	0.9843	0.9686	0.9685

Table S4. The ROC-AUC, precision, recall, F1, accuracy, MCC and Cohen´ Kappa scores for Random Forest (RF) classifier models for the train and validation datasets of endocrine disruption in TOX21 dataset using the 5-fold cross validation method. AR stands for androgen receptors, ER estrogen receptors, AhR aryl hydrocarbon receptors, ARO aromatase receptors, PPAR peroxisome proliferator–activated receptors.

**Task /Train**	**Scores**						
	**ROC-AUC**	**Precision**	**Recall**	**F1**	**Accuracy**	**MCC**	**Cohen´ Kappa**
**AR**	0.9997	0.9989	0.9374	0.9681	0.9681	0.9381	0.9363
**ER**	0.9807	0.9558	0.9126	0.9356	0.9356	0.8721	0.8712
**AhR**	0.9968	0.9542	0.9933	0.9728	0.9728	0.9465	0.9457
**ARO**	0.9994	0.9984	0.9704	0.9845	0.9845	0.9694	0.9690
**PPAR**	0.9998	0.9964	0.9832	0.9898	0.9898	0.9798	0.9797
**Task /Validation**	**Scores**						
	**ROC-AUC**	**Precision**	**Recall**	**F1**	**Accuracy**	**MCC**	**Cohen´ Kappa**
**AR**	0.9975	0.9908	0.9355	0.9636	0.9636	0.9286	0.9271
**ER**	0.9615	0.9195	0.8940	0.9055	0.9055	0.8115	0.8111
**AhR**	0.9902	0.9080	0.9895	0.9445	0.9445	0.8926	0.8890
**ARO**	0.9980	0.9911	0.9824	0.9865	0.9865	0.9731	0.9730
**PPAR**	0.9985	0.9894	0.9894	0.9882	0.9882	0.9764	0.9764

Table S5. The ROC-AUC, precision, recall, F1, accuracy, MCC and Cohen´ Kappa scores for Random Forest (RF) classifier models for the train and validation datasets of endocrine disruption in TOX21 dataset using the 10-fold cross validation method. AR stands for androgen receptors, ER estrogen receptors, AhR aryl hydrocarbon receptors, ARO aromatase receptors, PPAR peroxisome proliferator–activated receptors.

Task /Train	Scores						
	ROC-AUC	Precision	Recall	F1	Accuracy	MCC	Cohen´ Kappa
**AR**	0.9998	0.9991	0.9264	0.9631	0.9631	0.9285	0.9261
**ER**	0.9804	0.9539	0.9079	0.9319	0.9319	0.8649	0.8639
**AhR**	0.9972	0.9597	0.9950	0.9764	0.9764	0.9535	0.9528
**ARO**	0.9988	0.9989	0.9728	0.9859	0.9859	0.9721	0.9718
**PPAR**	0.9998	0.9982	0.9851	0.9917	0.9917	0.9835	0.9834
**Task /Validation**	**Scores**						
	**ROC-AUC**	**Precision**	**Recall**	**F1**	**Accuracy**	**MCC**	**Cohen´ Kappa**
**AR**	0.9987	0.9950	0.9148	0.9538	0.9538	0.9109	0.9077
**ER**	0.9551	0.9027	0.8806	0.8934	0.8934	0.7870	0.7867
**AhR**	0.9919	0.8949	0.9950	0.9412	0.9412	0.8878	0.8827
**ARO**	0.9948	0.9848	0.9710	0.9780	0.9780	0.9562	0.9561
**PPAR**	0.9986	0.9867	0.9789	0.9829	0.9829	0.9660	0.9659

Table S6. Summary of the chemical compounds classified by the classification models for the AR, ER, AhR, ARO, and PPAR targets. The application of each chemical compound is also presented. Comparison with literature for AR or ER acitivity.[1–5]

Model	Chemical compounds	Comparison	Target	Application
AR-EDC	1,2-Dibromo-4-(1,2-dibromoethyl)cyclohexane	N, S	AR[6–13]<br>ER[13]	Flame retardant
AR-EDKB	Epitestosterone	S[1,3]	AR[14]<br>ER[5,15]	Masking agent
	Progesterone	S[1,3]	AR[16]	Contraception
ER-EDC	Zearalenone	M[1]	ER[17–22]	Mycotoxin
	Quinalphos	P[1,3]	AC[23,24]<br>AR[3]<br>AhR[25]	Pesticide
	Propylparaben	P[1,2,4]	ER [26–31]	Preservative
	2-Ethylhexyl 4-hydroxybenzoate	P[1,2,4]	ER [26–31]	Food preservative
	Hydroxychlor	M, P[1,3,4]	AR[3]<br>ER[4,32,33]<br>ARO[34]	Pesticide metabolite
ER-EDKB	Epitestosterone	S[1,5]	AR[14]<br>ER[5,15]	Masking agent
	Estradiol	S[1,5]	ER[5,15]<br>ARO[35]	Contraception
	Ethinyl estradiol	S[1,5]	ER[36,37]	Contraception
	Dihydrotestosterone	S[1,5]	AR[38]<br>ER[39,40]	Steroid
	5α-androstane-3α,17β-diol	S[1,5]	AR[41,42]<br>ER[43,44]	Steroid metabolite
	Progesterone	S[1,5]	AR[16]<br>ER[45–48]	Contraception
AhR-EDC	Profenofos	P[1,3]	AC[23,24,49]<br>AR[3]<br>AhR[25,50]	Insecticide
	Triadimefon	V[2]	AC[23,24]	Fungicide
	Isofenphos	N, P[1]	AC[23,24,51]<br>AR[50,52]<br>ER[50,52]	Insecticide
	Cybutryne	V[1,3]	AR[1,3]	Insecticide
	Methyl parathion	P[1,3]	AC[23,24]<br>AR[1,3,50,52]	Pesticide
	Bupirimate	P[2]	PXR[2,53]	Fungicide
AhR-EDKB	Eucalyptol	N	AR[54,55]<br>ER[54,55]<br>AhR[56]	Flavorings, fragrances, and cosmetics

	Carbaryl	X[2]	AC[23,24]<br>ER[57]<br>AhR[50,52,58,59]	Insecticide
	Propanil	TP[2], low AR activity (FP)[1]	AC[23,24]<br>AR[50,52]<br>ER[60]<br>AhR[25,50,52]	Herbicide
	Parathion	133, 135	AC[23,24]<br>AR[50,52]<br>AhR[61]	Insecticide
	Melatonin	X[2]	AR[62]<br>ER[63]<br>AhR[64]	Sleep disorder
	Linuron	FP[1]	AC[23,24]<br>AR[50,52]<br>AhR[25,50,52]	Herbicide
ARO-EDC	Forskolin	N, S	AR[65]<br>ER[66]<br>ARO[67]	Heart failure, fat loss
	Chlorfenvinfos	P[1]	AC[23,24]<br>AR[1]<br>ER[68]	Insecticide
	Methyl paraoxon	M, P[1]	AR[1]<br>AC[23,24,51]	Insecticide
	Tolylfluanid	N, P[2]	PPAR[69]	Fungicide
	Enilconazole	V[1]	AR[70]<br>ER[70]<br>ARO[70]	Fungicide
ARO-EDKB	S-fulvestrant	N, S, V[1,71]	AR[72]<br>ER[72]<br>ARO[73,74]	Cancer
	Estradiol	S[1]	ER<br>ARO[35,75]	Contraception
	Tamoxifen	N, V[1]	AR[76]<br>ER[77]<br>ARO[78]	Cancer
PPAR-EDC	2-(thiocyanomethylthio)benzothiazole	N, V[2]	PPAR[79]	Preservative<br>Biocide<br>Fungicide

X: structural alerts found in literature; M: structural alerts not found because the active compound is a metabolite; N: new structural alerts; P: piece of structural alert is found; V: structural alert is found at vicinity; S: several structural alerts encompass most of the molecule. AC: acetylcholinesterase activity; PXR: Pregnane X cellular receptor.

References

(1) Nendza, M.; Wenzel, A.; Müller, M.; Lewin, G.; Simetska, N.; Stock, F.; Arning, J. Screening for Potential Endocrine Disruptors in Fish: Evidence from Structural Alerts and in Vitro and in Vivo Toxicological Assays. *Environ Sci Eur* **2016**, *28* (1), 26. https://doi.org/10.1186/s12302-016-0094-5.

(2) Zhang, R.; Wang, B.; Li, L.; Li, S.; Guo, H.; Zhang, P.; Hua, Y.; Cui, X.; Li, Y.; Mu, Y.; Huang, X.; Li, X. Modeling and Insights into the Structural Characteristics of Endocrine-Disrupting Chemicals. *Ecotoxicol Environ Saf* **2023**, *263*, 115251. https://doi.org/10.1016/j.ecoenv.2023.115251.

(3) Fang, H.; Tong, W.; Branham, W. S.; Moland, C. L.; Dial, S. L.; Hong, H.; Xie, Q.; Perkins, R.; Owens, W.; Sheehan, D. M. Study of 202 Natural, Synthetic, and Environmental Chemicals for Binding to the Androgen Receptor. *Chem Res Toxicol* **2003**, *16* (10), 1338–1358. https://doi.org/10.1021/tx030011g.

(4) Blair, R. M. The Estrogen Receptor Relative Binding Affinities of 188 Natural and Xenochemicals: Structural Diversity of Ligands. *Toxicol. Sci.* **2000**, *54* (1), 138–153. https://doi.org/10.1093/toxsci/54.1.138.

(5) Hong, H.; Tong, W.; Fang, H.; Shi, L.; Xie, Q.; Wu, J.; Perkins, R.; Walker, J. D.; Branham, W.; Sheehan, D. M. Prediction of Estrogen Receptor Binding for 58,000 Chemicals Using an Integrated System of a Tree-Based Model with Structural Alerts. *Environ Health Perspect* **2002**, *110* (1), 29–36. https://doi.org/10.1289/ehp.0211029.

(6) Khalaf, H.; Larsson, A.; Berg, H.; McCrindle, R.; Arsenault, G.; Olsson, P.-E. Diastereomers of the Brominated Flame Retardant 1,2-Dibromo-4-(1,2 Dibromoethyl)Cyclohexane Induce Androgen Receptor Activation in the HepG2 Hepatocellular Carcinoma Cell Line and the LNCaP Prostate Cancer Cell Line. *Environ Health Perspect* **2009**, *117* (12), 1853–1859. https://doi.org/10.1289/ehp.0901065.

(7) Curran, I. H. A.; Liston, V.; Nunnikhoven, A.; Caldwell, D.; Scuby, M. J. S.; Pantazopoulos, P.; Rawn, D. F. K.; Coady, L.; Armstrong, C.; Lefebvre, D. E.; Bondy, G. S. Toxicologic Effects of 28-Day Dietary Exposure to the Flame Retardant 1,2-Dibromo-4-(1,2-Dibromoethyl)-Cyclohexane (TBECH) in F344 Rats. *Toxicology* **2017**, *377*, 1–13. https://doi.org/10.1016/j.tox.2016.12.001.

(8) Kharlyngdoh, J. B.; Pradhan, A.; Olsson, P.-E. Androgen Receptor Modulation Following Combination Exposure to Brominated Flame-Retardants. *Sci Rep* **2018**, *8* (1), 4843. https://doi.org/10.1038/s41598-018-23181-0.

(9) Kharlyngdoh, J. B.; Asnake, S.; Pradhan, A.; Olsson, P.-E. TBECH, 1,2-Dibromo-4-(1,2 Dibromoethyl) Cyclohexane, Alters Androgen Receptor Regulation in Response to Mutations Associated with Prostate Cancer. *Toxicol Appl Pharmacol* **2016**, *307*, 91–101. https://doi.org/10.1016/j.taap.2016.07.018.

(10) Pradhan, A.; Asnake, S.; Kharlyngdoh, J. B.; Modig, C.; Olsson, P.-E. In Silico and Biological Analysis of Anti-Androgen Activity of the Brominated Flame Retardants ATE, BATE and DPTE in Zebrafish. *Chem Biol Interact* **2015**, *233*, 35–45. https://doi.org/10.1016/j.cbi.2015.03.023.

(11) Wong, L. I. L.; Reers, A. R.; Currier, H. A.; Williams, T. D.; Cox, M. E.; Elliott, J. E.; Beischlag, T. V. The Effects of the Organic Flame-Retardant

1,2-Dibromo-4-(1,2-dibromoethyl) Cyclohexane (TBECH) on Androgen Signaling in Human Prostate Cancer Cell Lines. *J Biochem Mol Toxicol* **2016**, *30* (5), 239–242. https://doi.org/10.1002/jbt.21784.

(12) Larsson, A.; Eriksson, L. A.; Andersson, P. L.; Ivarson, P.; Olsson, P.-E. Identification of the Brominated Flame Retardant 1,2-Dibromo-4-(1,2-Dibromoethyl)Cyclohexane as an Androgen Agonist. *J Med Chem* **2006**, *49* (25), 7366–7372. https://doi.org/10.1021/jm060713d.

(13) Asnake, S.; Pradhan, A.; Banjop-Kharlyngdoh, J.; Modig, C.; Olsson, P. 1,2-dibromo-4-(1,2 Dibromoethyl) Cyclohexane (TBECH)–Mediated Steroid Hormone Receptor Activation and Gene Regulation in Chicken LMH Cells. *Environ Toxicol Chem* **2014**, *33* (4), 891–899. https://doi.org/10.1002/etc.2509.

(14) Stárka, L.; Bičíková, M.; Hampl, R. Epitestosterone—an Endogenous Antiandrogen? *J Steroid Biochem* **1989**, *33* (5), 1019–1021. https://doi.org/10.1016/0022-4731(89)90255-0.

(15) Bicíková, M.; Klak, J.; Hill, M.; Stárka, L. The Effect of Epitestosterone on Estrogen Biosynthesis in Vitro. *Horm Metab Res* **2000**, *32* (4), 125–128.

(16) O’Shaughnessy, P. J.; Antignac, J. P.; Le Bizec, B.; Morvan, M.-L.; Svechnikov, K.; Söder, O.; Savchuk, I.; Monteiro, A.; Soffientini, U.; Johnston, Z. C.; Bellingham, M.; Hough, D.; Walker, N.; Filis, P.; Fowler, P. A. Alternative (Backdoor) Androgen Production and Masculinization in the Human Fetus. *PLoS Biol* **2019**, *17* (2), e3000002. https://doi.org/10.1371/journal.pbio.3000002.

(17) Ropejko, K.; Twarużek, M. Zearalenone and Its Metabolites—General Overview, Occurrence, and Toxicity. *Toxins (Basel)* **2021**, *13* (1), 35. https://doi.org/10.3390/toxins13010035.

(18) Chi, M. S.; Mirocha, C. J.; Weaver, G. A.; Kurtz, H. J. Effect of Zearalenone on Female White Leghorn Chickens. *Appl Environ Microbiol* **1980**, *39* (5), 1026–1030. https://doi.org/10.1128/aem.39.5.1026-1030.1980.

(19) Pompa, G.; Montesissa, C.; Di Lauro, F. M.; Fadini, L. The Metabolism of Zearalenone in Subcellular Fractions from Rabbit and Hen Hepatocytes and Its Estrogenic Activity in Rabbits. *Toxicology* **1986**, *42* (1), 69–75. https://doi.org/10.1016/0300-483X(86)90093-4.

(20) Metzler, M.; Pfeiffer, E.; Hildebrand, A. Zearalenone and Its Metabolites as Endocrine Disrupting Chemicals. *World Mycotoxin J* **2010**, *3* (4), 385–401. https://doi.org/10.3920/WMJ2010.1244.

(21) Lecomte, S.; Demay, F.; Pham, T. H.; Moulis, S.; Efstathiou, T.; Chalmel, F.; Pakdel, F. Deciphering the Molecular Mechanisms Sustaining the Estrogenic Activity of the Two Major Dietary Compounds Zearalenone and Apigenin in ER-Positive Breast Cancer Cell Lines. *Nutrients* **2019**, *11* (2), 237. https://doi.org/10.3390/nu11020237.

(22) Zinedine, A.; Soriano, J. M.; Moltó, J. C.; Mañes, J. Review on the Toxicity, Occurrence, Metabolism, Detoxification, Regulations and Intake of Zearalenone: An Oestrogenic Mycotoxin. *Food Chem. Toxicol.* **2007**, *45* (1), 1–18. https://doi.org/10.1016/j.fct.2006.07.030.

(23) Schug, T. T.; Blawas, A. M.; Gray, K.; Heindel, J. J.; Lawler, C. P. Elucidating the Links Between Endocrine Disruptors and Neurodevelopment. *Endocrinology* **2015**, *156* (6), 1941–1951. https://doi.org/10.1210/en.2014-1734.

(24) Patisaul, H. B. Endocrine Disruption of Vasopressin Systems and Related Behaviors. *Front Endocrinol (Lausanne)* **2017**, *8*. https://doi.org/10.3389/fendo.2017.00134.

(25) Takeuchi, S.; Iida, M.; Yabushita, H.; Matsuda, T.; Kojima, H. In Vitro Screening for Aryl Hydrocarbon Receptor Agonistic Activity in 200 Pesticides Using a Highly Sensitive Reporter Cell Line, DR-EcoScreen Cells, and in Vivo Mouse Liver Cytochrome P450-1A Induction by Propanil, Diuron and Linuron. *Chemosphere* **2008**, *74* (1), 155–165. https://doi.org/10.1016/j.chemosphere.2008.08.015.

(26) Martín, J. M. P.; Freire, P. F.; Daimiel, L.; Martínez-Botas, J.; Sánchez, C. M.; Lasunción, M. Á.; Peropadre, A.; Hazen, M. J. The Antioxidant Butylated Hydroxyanisole Potentiates the Toxic Effects of Propylparaben in Cultured Mammalian Cells. *Food Chem. Toxicol.* **2014**, *72*, 195–203. https://doi.org/10.1016/j.fct.2014.07.031.

(27) Klopčič, I.; Kolšek, K.; Dolenc, M. S. Glucocorticoid-like Activity of Propylparaben, Butylparaben, Diethylhexyl Phthalate and Tetramethrin Mixtures Studied in the MDA-Kb2 Cell Line. *Toxicol Lett* **2015**, *232* (2), 376–383. https://doi.org/10.1016/j.toxlet.2014.11.019.

(28) Jiménez-Díaz, I.; Artacho-Cordón, F.; Vela-Soria, F.; Belhassen, H.; Arrebola, J. P.; Fernández, M. F.; Ghali, R.; Hedhili, A.; Olea, N. Urinary Levels of Bisphenol A, Benzophenones and Parabens in Tunisian Women: A Pilot Study. *Sci. Total Environ.* **2016**, *562*, 81–88. https://doi.org/10.1016/j.scitotenv.2016.03.203.

(29) Gazin, V.; Marsden, E.; Marguerite, F. Oral Propylparaben Administration to Juvenile Male Wistar Rats Did Not Induce Toxicity in Reproductive Organs. *Toxicol. Sci.* **2013**, *136* (2), 392–401. https://doi.org/10.1093/toxsci/kft211.

(30) Chen, Y.; Zhao, C.; Zheng, J.; Su, N.; Ji, H. Discovery of the Mechanism of N-Propylparaben-Promoting the Proliferation of Human Breast Adenocarcinoma Cells by Activating Human Estrogen Receptors via Metabolomics Analysis. *Hum Exp Toxicol* **2023**, *42*, 096032712311716. https://doi.org/10.1177/09603271231171648.

(31) Matten, S.; Fallacara, D.; Kamel, A.; Lynn, S. G.; Fort, D. J.; Wolf, J. C.; Leak, T.; von Holst, H.; Bever, R. J.; Thomas, A.; Brown, V. J. Evaluation of Multigenerational Effects of 2-ethylhexyl 4-hydroxybenzoate in Japanese Medaka. *J. Appl. Toxicol.* **2023**, *43* (11), 1645–1666. https://doi.org/10.1002/jat.4502.

(32) Boberg, J.; Taxvig, C.; Christiansen, S.; Hass, U. Possible Endocrine Disrupting Effects of Parabens and Their Metabolites. *Reprod. Toxicol.* **2010**, *30* (2), 301–312. https://doi.org/10.1016/j.reprotox.2010.03.011.

(33) Bolger, R.; Wiese, T. E.; Ervin, K.; Nestich, S.; Checovich, W. Rapid Screening of Environmental Chemicals for Estrogen Receptor Binding Capacity. *Environ Health Perspect* **1998**, *106* (9), 551–557. https://doi.org/10.1289/ehp.98106551.

(34) Liu, S.; Mao, B.; Bai, Y.; Liu, J.; Li, H.; Li, X.; Lian, Q.; Ge, R.-S. Effects of Methoxychlor and Its Metabolite Hydroxychlor on Human Placental 3β-Hydroxysteroid Dehydrogenase 1 and Aromatase in JEG-3 Cells. *Pharmacology* **2016**, *97* (3–4), 126–133. https://doi.org/10.1159/000442711.

(35) Bose, H. S.; Whittal, R. M.; Lanier, C. E.; Marshall, B.; Rajapaksha, M.; Wheeler, B. W.; Carbo, N. D.; Hahn, E. M.; Perry, E. W.; Hall, N. M.; Melomed, M. M.; Perkins, E. L.; Burak, W. E. Regulation of Estradiol Synthesis by Aromatase Interacting Partner in Breast (AIPB). *Mol Cell Biol* **2021**, *41* (11). https://doi.org/10.1128/MCB.00357-21.

(36) Kuhl, H. Pharmacology of Estrogens and Progestogens: Influence of Different Routes of Administration. *Climacteric* **2005**, *8* (sup1), 3–63. https://doi.org/10.1080/13697130500148875.

(37) Escande, A.; Pillon, A.; Servant, N.; Cravedi, J.-P.; Larrea, F.; Muhn, P.; Nicolas, J.-C.; Cavaillès, V.; Balaguer, P. Evaluation of Ligand Selectivity Using Reporter Cell Lines Stably Expressing Estrogen Receptor Alpha or Beta. *Biochem Pharmacol* **2006**, *71* (10), 1459–1469. https://doi.org/10.1016/j.bcp.2006.02.002.

(38) Grino, P. B.; Griffin, J. E.; Wilson, J. D. Testosterone at High Concentrations Interacts with the Human Androgen Receptor Similarly to Dihydrotestosterone. *Endocrinology* **1990**, *126* (2), 1165–1172. https://doi.org/10.1210/endo-126-2-1165.

(39) Horton, A. C.; Wilkinson, M. M.; Kilanowski-Doroh, I.; Dong, Z.; Liu, J.; Ogola, B. O.; Visniauskas, B.; Lindsey, S. H. Dihydrotestosterone Induces Arterial Stiffening in Female Mice. *Biol Sex Differ* **2024**, *15* (1), 9. https://doi.org/10.1186/s13293-024-00586-3.

(40) Conway, B.-A.; Mills, T. M. In Vitro Effects of Dihydrotestosterone on Granulosa Cell Production of Estrogen and Progesterone. *Steroids* **1991**, *56* (5), 258–262. https://doi.org/10.1016/0039-128X(91)90044-V.

(41) Penning, T. M.; Bauman, D. R.; Jin, Y.; Rizner, T. L. Identification of the Molecular Switch That Regulates Access of 5α-DHT to the Androgen Receptor. *Mol Cell Endocrinol* **2007**, *265–266*, 77–82. https://doi.org/10.1016/j.mce.2006.12.007.

(42) Jin, Y.; Penning, T. M. Steroid 5α-Reductases and 3α-Hydroxysteroid Dehydrogenases: Key Enzymes in Androgen Metabolism. *Best Pract Res Clin Endocrinol Metab* **2001**, *15* (1), 79–94. https://doi.org/10.1053/beem.2001.0120.

(43) Kuiper, G. G. J. M.; Carlsson, B.; Grandien, K.; Enmark, E.; Häggblad, J.; Nilsson, S.; Gustafsson, J.-A. Comparison of the Ligand Binding Specificity and Transcript Tissue Distribution of Estrogen Receptors α and β. *Endocrinology* **1997**, *138* (3), 863–870. https://doi.org/10.1210/endo.138.3.4979.

(44) Baker, M. Recent Insights into the Origins of Adrenal and Sex Steroid Receptors. *J Mol Endocrinol* **2002**, *28* (3), 149–152. https://doi.org/10.1677/jme.0.0280149.

(45) Aufrère, M. B.; Benson, H. Progesterone: An Overview and Recent Advances. *J Pharm Sci* **1976**, *65* (6), 783–800. https://doi.org/10.1002/jps.2600650602.

(46) Mohammed, H.; Russell, I. A.; Stark, R.; Rueda, O. M.; Hickey, T. E.; Tarulli, G. A.; Serandour, A. A.; Birrell, S. N.; Bruna, A.; Saadi, A.; Menon, S.; Hadfield, J.; Pugh, M.; Raj, G. V.; Brown, G. D.; D'Santos, C.; Robinson, J. L. L.; Silva, G.; Launchbury, R.; Perou, C. M.; Stingl, J.; Caldas, C.; Tilley, W. D.; Carroll, J. S. Progesterone Receptor Modulates ERα Action in Breast Cancer. *Nature* **2015**, *523* (7560), 313–317. https://doi.org/10.1038/nature14583.

(47) Ellmann, S.; Sticht, H.; Thiel, F.; Beckmann, M. W.; Strick, R.; Strissel, P. L. Estrogen and Progesterone Receptors: From Molecular Structures to Clinical Targets. *Cell. Mol. Life Sci.* **2009**, *66* (15), 2405–2426. https://doi.org/10.1007/s00018-009-0017-3.

(48) Jayaraman, A.; Pike, C. J. Progesterone Attenuates Oestrogen Neuroprotection Via Downregulation of Oestrogen Receptor Expression in Cultured Neurones. *J Neuroendocrinol* **2009**, *21* (1), 77–81. https://doi.org/10.1111/j.1365-2826.2008.01801.x.

(49) Sultana, Z.; Khan, Mst. M.; Mostakim, G. M.; Moniruzzaman, Md.; Rahman, Md. K.; Shahjahan, Md.; Islam, M. S. Studying the Effects of Profenofos, an Endocrine Disruptor, on Organogenesis of Zebrafish. *Environ. Sci. Pollut. Res. Int.* **2021**, *28* (16), 20659–20667. https://doi.org/10.1007/s11356-020-11944-0.

(50) Kojima, H.; Takeuchi, S.; Nagai, T. Endocrine-Disrupting Potential of Pesticides via Nuclear Receptors and Aryl Hydrocarbon Receptor. *J. Health Sci.* **2010**, *56* (4), 374–386. https://doi.org/10.1248/jhs.56.374.

(51) Henderson, J. D.; Higgins, R. J.; Dacre, J. C.; Wilson, B. W. Neurotoxicity of Acute and Repeated Treatments of Tabun, Paraoxon, Diisopropyl Fluorophosphate and Isofenphos to the Hen. *Toxicology* **1992**, *72* (2), 117–129. https://doi.org/10.1016/0300-483X(92)90106-O.

(52) Teraoka, H.; Dong, W.; Okuhara, Y.; Iwasa, H.; Shindo, A.; Hill, A. J.; Kawakami, A.; Hiraga, T. Impairment of Lower Jaw Growth in Developing Zebrafish Exposed to 2,3,7,8-Tetrachlorodibenzo-p-Dioxin and Reduced Hedgehog Expression. *Aquat. Toxicol.* **2006**, *78* (2), 103–113. https://doi.org/10.1016/j.aquatox.2006.02.009.

(53) Lemaire, G.; Mnif, W.; Pascussi, J.-M.; Pillon, A.; Rabenoelina, F.; Fenet, H.; Gomez, E.; Casellas, C.; Nicolas, J.-C.; Cavaillès, V.; Duchesne, M.-J.; Balaguer, P. Identification of New Human Pregnane X Receptor Ligands among Pesticides Using a Stable Reporter Cell System. *Toxicol. Sci.* **2006**, *91* (2), 501–509. https://doi.org/10.1093/toxsci/kfj173.

(54) Ramsey, J. T.; Li, Y.; Arao, Y.; Naidu, A.; Coons, L. A.; Diaz, A.; Korach, K. S. Lavender Products Associated With Premature Thelarche and Prepubertal Gynecomastia: Case Reports and Endocrine-Disrupting Chemical Activities. *J Clin Endocrinol Metab* **2019**, *104* (11), 5393–5405. https://doi.org/10.1210/jc.2018-01880.

(55) Liu, H.; Sun, W.; Zhu, H.; Guo, J.; Liu, M.; Xu, S. Eucalyptol Relieves the Toxicity of Diisobutyl Phthalate in Ctenopharyngodon Idellus Kidney Cells through Keap1/Nrf2/HO-1 Pathway: Apoptosis-Autophagy Crosstalk and Immunoregulation. *Fish Shellfish Immunol* **2022**, *130*, 490–500. https://doi.org/10.1016/j.fsi.2022.09.056.

(56) Lee, J.; Ha, S. J.; Park, J.; Kim, Y. H.; Lee, N. H.; Kim, Y. E.; Kim, Y.; Song, K.-M.; Jung, S. K. 1,8-Cineole Prevents UVB-Induced Skin Carcinogenesis by Targeting the Aryl Hydrocarbon Receptor. *Oncotarget* **2017**, *8* (62), 105995–106008. https://doi.org/10.18632/oncotarget.22519.

(57) Mnif, W.; Hassine, A. I. H.; Bouaziz, A.; Bartegi, A.; Thomas, O.; Roig, B. Effect of Endocrine Disruptor Pesticides: A Review. *Int J Environ Res Public Health* **2011**, *8* (6), 2265–2303. https://doi.org/10.3390/ijerph8062265.

(58) Casado, S.; Alonso, M.; Herradón, B.; Tarazona, J. V.; Navas, J. M. Activation of the Aryl Hydrocarbon Receptor by Carbaryl: Computational Evidence of the Ability of Carbaryl to Assume a Planar Conformation. *Environ Toxicol Chem* **2006**, *25* (12), 3141–3147. https://doi.org/10.1897/06-131R.1.

(59) Denison, M. S.; Phelan, D.; Winter, G. M.; Ziccardi, M. H. Carbaryl, a Carbamate Insecticide, Is a Ligand for the Hepatic Ah (Dioxin) Receptor. *Toxicol Appl Pharmacol* **1998**, *152* (2), 406–414. https://doi.org/10.1006/taap.1998.9999.

(60) Salazar, K. D.; Miller, M. R.; Barnett, J. B.; Schafer, R. Evidence for a Novel Endocrine Disruptor: The Pesticide Propanil Requires the Ovaries and Steroid Synthesis to Enhance Humoral Immunity. *Toxicol. Sci.* **2006**, *93* (1), 62–74. https://doi.org/10.1093/toxsci/kfl038.

(61) Vrzal, R.; Zenata, O.; Doricakova, A.; Dvorak, Z. Environmental Pollutants Parathion, Paraquat and Bisphenol A Show Distinct Effects towards Nuclear Receptors-Mediated Induction of Xenobiotics-Metabolizing Cytochromes P450 in Human Hepatocytes. *Toxicol Lett* **2015**, *238* (1), 43–53. https://doi.org/10.1016/j.toxlet.2015.07.008.

(62) Rimler, A.; Culig, Z.; Lupowitz, Z.; Zisapel, N. Nuclear Exclusion of the Androgen Receptor by Melatonin. *J Steroid Biochem Mol Biol* **2002**, *81* (1), 77–84. https://doi.org/10.1016/S0960-0760(02)00050-X.

(63) Cos, S.; Gonzalez, A.; Martinez-Campa, C.; Mediavilla, M.; Alonso-Gonzalez, C.; Sanchez-Barcelo, E. Melatonin as a Selective Estrogen Enzyme Modulator. *Curr Cancer Drug Targets* **2008**, *8* (8), 691–702. https://doi.org/10.2174/156800908786733469.

(64) Slominski, A. T.; Kim, T.-K.; Slominski, R. M.; Song, Y.; Qayyum, S.; Placha, W.; Janjetovic, Z.; Kleszczyński, K.; Atigadda, V.; Song, Y.; Raman, C.; Elferink, C. J.; Hobrath, J. V.; Jetten, A. M.; Reiter, R. J. Melatonin and Its Metabolites Can Serve as Agonists on the Aryl Hydrocarbon Receptor and Peroxisome Proliferator-Activated Receptor Gamma. *Int J Mol Sci* **2023**, *24* (20), 15496. https://doi.org/10.3390/ijms242015496.

(65) Blok, L. J.; de Ruiter, P. E.; Brinkmann, A. O. Forskolin-Induced Dephosphorylation of the Androgen Receptor Impairs Ligand Binding. *Biochemistry* **1998**, *37* (11), 3850–3857. https://doi.org/10.1021/bi9724422.

(66) Tsai, H.-W.; Lin, V. Y.; Shupnik, M. A. Forskolin Stimulates Estrogen Receptor (ER) α Transcriptional Activity and Protects ER from Degradation by Distinct Mechanisms. *Int J Endocrinol* **2022**, *2022*, 1–17. https://doi.org/10.1155/2022/7690166.

(67) Sanderson, J. T. Induction and Inhibition of Aromatase (CYP19) Activity by Natural and Synthetic Flavonoid Compounds in H295R Human Adrenocortical Carcinoma Cells. *Toxicol. Sci.* **2004**, *82* (1), 70–79. https://doi.org/10.1093/toxsci/kfh257.

(68) Vinggaard, A. M.; Breinholt, V.; Larsen, J. C. Screening of Selected Pesticides for Oestrogen Receptor Activation in Vitro. *Food Addit Contam* **1999**, *16* (12), 533–542. https://doi.org/10.1080/026520399283678.

(69) Regnier, S. M.; Kirkley, A. G.; Ye, H.; El-Hashani, E.; Zhang, X.; Neel, B. A.; Kamau, W.; Thomas, C. C.; Williams, A. K.; Hayes, E. T.; Massad, N. L.; Johnson, D. N.; Huang, L.; Zhang, C.; Sargis, R. M. Dietary Exposure to the Endocrine Disruptor Tolylfluanid Promotes Global Metabolic Dysfunction in Male Mice. *Endocrinology* **2015**, *156* (3), 896–910. https://doi.org/10.1210/en.2014-1668.

(70) Jin, C.; Zhang, R.; Fu, Z.; Jin, Y. Maternal Exposure to Imazalil Disrupts the Endocrine System in F1 Generation Mice. *Mol Cell Endocrinol* **2019**, *486*, 105–112. https://doi.org/10.1016/j.mce.2019.03.002.

(71) Skórka-Majewicz, M.; Goschorska, M.; Żwierełło, W.; Baranowska-Bosiacka, I.; Styburski, D.; Kapczuk, P.; Gutowska, I. Effect of Fluoride on Endocrine Tissues and Their Secretory Functions -- Review. *Chemosphere* **2020**, *260*, 127565. https://doi.org/10.1016/j.chemosphere.2020.127565.

(72) Lai, A. C.; Crews, C. M. Induced Protein Degradation: An Emerging Drug Discovery Paradigm. *Nat Rev Drug Discov* **2017**, *16* (2), 101–114. https://doi.org/10.1038/nrd.2016.211.

(73) Sammons, S.; Kornblum, N. S.; Blackwell, K. L. Fulvestrant-Based Combination Therapy for Second-Line Treatment of Hormone Receptor-Positive Advanced Breast Cancer. *Target Oncol* **2019**, *14* (1), 1–12. https://doi.org/10.1007/s11523-018-0587-9.

(74) Teodoro, M. I.; Mayer, A.; da Costa Miranda, A.; Nunes, H.; da Costa, F. A.; Lourenço, A. Real-World Effectiveness of Aromatase Inhibitors and Fulvestrant in HR+/HER2- Advanced Breast Cancer: A Snapshot of the Last Two Years before Conventional Use of CDK 4/6 Inhibitors in a Portuguese Institution. *J Pharm Policy Pract* **2024**, *17* (1). https://doi.org/10.1080/20523211.2023.2296551.

(75) Pasqualini, J. R.; Chetrite, G. S. Estradiol as an Anti-Aromatase Agent in Human Breast Cancer Cells. *J Steroid Biochem Mol Biol* **2006**, *98* (1), 12–17. https://doi.org/10.1016/j.jsbmb.2005.10.001.

(76) Viani, G. A.; Bernardes da Silva, L. G.; Stefano, E. J. Prevention of Gynecomastia and Breast Pain Caused by Androgen Deprivation Therapy in Prostate Cancer: Tamoxifen or Radiotherapy? *Int. J. Radiat. Oncol. Biol. Phys.* **2012**, *83* (4), e519–e524. https://doi.org/10.1016/j.ijrobp.2012.01.036.

(77) Loi, S.; Criscitiello; Fumagalli; Saini. Tamoxifen in Early-Stage Estrogen Receptor-Positive Breast Cancer: Overview of Clinical Use and Molecular Biomarkers for Patient Selection. *Onco Targets Ther* **2010**, *4*, 1-11. https://doi.org/10.2147/OTT.S10155.

(78) Catalano, S.; Giordano, C.; Panza, S.; Chemi, F.; Bonofiglio, D.; Lanzino, M.; Rizza, P.; Romeo, F.; Fuqua, S. A. W.; Maggiolini, M.; Andò, S.; Barone, I. Tamoxifen through GPER Upregulates Aromatase Expression: A Novel Mechanism Sustaining Tamoxifen-Resistant Breast Cancer Cell Growth. *Breast Cancer Res Treat* **2014**, *146* (2), 273–285. https://doi.org/10.1007/s10549-014-3017-4.

(79) Azam, M. A. Biological Activities of 2-Mercaptobenzothiazole Derivatives: A Review. *Sci Pharm* **2012**, *80* (4), 789–823. https://doi.org/10.3797/scipharm.1204-27.
